# Stretchable Piezoresistive Pressure Sensor Array with Sophisticated Sensitivity, Strain‐Insensitivity, and Reproducibility

**DOI:** 10.1002/advs.202405374

**Published:** 2024-07-16

**Authors:** Su Bin Choi, Taejoon Noh, Seung‐Boo Jung, Jong‐Woong Kim

**Affiliations:** ^1^ Department of Smart Fab Technology Sungkyunkwan University Suwon 16419 South Korea; ^2^ School of Advanced Materials Science and Engineering Sungkyunkwan University Suwon 16419 South Korea; ^3^ Department of Semiconductor Convergence Engineering Sungkyunkwan University Suwon 16419 South Korea; ^4^ School of Mechanical Engineering Sungkyunkwan University Suwon 16419 South Korea

**Keywords:** deep‐learning, electrospinning, pressure sensor, strain‐insensitive, Ti_3_C_2_T_X_ MXene

## Abstract

This study delves into the development of a novel 10 by 10 sensor array featuring 100 pressure sensor pixels, achieving remarkable sensitivity up to 888.79 kPa^−1^, through the innovative design of sensor structure. The critical challenge of strain sensitivity inherent is addressed in stretchable piezoresistive pressure sensors, a domain that has seen significant interest due to their potential for practical applications. This approach involves synthesizing and electrospinning polybutadiene‐urethane (PBU), a reversible cross‐linking polymer, subsequently coated with MXene nanosheets to create a conductive fabric. This fabrication technique strategically enhances sensor sensitivity by minimizing initial current values and incorporating semi‐cylindrical electrodes with Ag nanowires (AgNWs) selectively coated for optimal conductivity. The application of a pre‐strain method to electrode construction ensures strain immunity, preserving the sensor's electrical properties under expansion. The sensor array demonstrated remarkable sensitivity by consistently detecting even subtle airflow from an air gun in a wind sensing test, while a novel deep learning methodology significantly enhanced the long‐term sensing accuracy of polymer‐based stretchable mechanical sensors, marking a major advancement in sensor technology. This research presents a significant step forward in enhancing the reliability and performance of stretchable piezoresistive pressure sensors, offering a comprehensive solution to their current limitations.

## Graphical abstract

A strain‐resistant, stretchable piezoresistive pressure sensor is engineered, featuring high sensitivity due to its semi‐cylindrical electrode structure and fiber‐based sensors. This innovative pressure sensor array boasts a smart electrode‐sensor configuration, offering sophisticated sensitivity and promising to revolutionize long‐term sensor reliability with extensive spatial resolution across a myriad of applications.

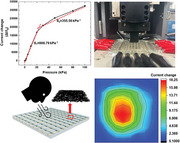

## 1 Introduction

The domain of stretchable piezoresistive pressure sensors has attracted considerable interest from the scientific community, owing to their inherently intuitive sensing mechanisms, simplistic architectural and manufacturing methodologies, and pronounced sensitivity under conditions of minimal pressure.^[^ ^1^, ^2^, ^3^, ^4^ ^]^ In contrast to their piezocapacitive and triboelectric counterparts, which are significantly susceptible to external variables such as ambient humidity, temperature fluctuations, and the proximity to conductive and magnetic substances, piezoresistive variants stand out for their enhanced suitability in practical deployments. Paramount among the criteria that determine the efficacy of pressure sensors are sensitivity, linearity, the breadth of detection range, and the upper limits of sensitivity, with the former being indispensable for ensuring the requisite levels of measurement reliability and precision.^[^ ^5^, ^6^, ^7^ ^]^ Accordingly, the augmentation of sensitivity and the bolstering of reliability in stretchable piezoresistive pressure sensors have been prioritized in recent investigative endeavors.^[^ ^8^, ^9^ ^]^ Furthermore, these sensors, by design, generate electrical signals that alter in accordance to strain, thus complicating the discernment of signals attributed solely to pressure variations.^[^ ^10^, ^11^, ^12^ ^]^ Compounded by the fact that these sensors are constituted of mechanically soft materials, rendering them susceptible to deformation under external forces, the independent detection of pressure becomes a complex challenge due to the concomitant influence of diverse stimuli.^[^ ^13^, ^14^ ^]^ Hence, there emerges an exigent demand for the innovation of sensors that not only excel in sensitivity but are also characterized by their resistance to strain‐induced distortions.

Efforts to augment the sensitivity of pressure sensors through the induction of shape deformation in conductive layers or the fabrication of intricate microstructures have been acknowledged as efficacious strategies.^[^ ^15^, ^16^, ^17^ ^]^ The operational efficacy of piezoresistive pressure sensors is fundamentally contingent upon the deformation of their conductive layers under external forces, which precipitates variations in the internal electrical signals. Consequently, the sensor's performance is significantly predicated on the architecture of the effective conductive layer.^[^ ^18^ ^]^ Numerous investigations have underscored the benefits of enhancing the surface modification of piezoresistive pressure sensors to achieve elevated sensitivity levels. Notably, sensitivity enhancements have been realized through the artificial fabrication of pressure sensors endowed with meticulously designed micro‐sized pyramidal^[^ ^19^ ^]^ or hollow sphere structures.^[^ ^20^ ^]^ This has been achieved either by the application of a conductive layer on a textured surface facilitated by sandpaper or through the development of templates. Moreover, the adoption of strategies aimed at diversifying and complexifying the structure by altering the electrode surface with natural bio‐templates, such as rose petals^[^ ^21^ ^]^ and mimosa leaves,^[^ ^22^ ^]^ has proven efficacious in attaining high sensitivity. However, the utility of leveraging these topographical modifications is inherently constrained to surface‐level improvements within the entire layer, thus limiting their impact on the sensitivity of the pressure response. A more advanced methodology involves utilizing the conductive layer, structured in its entirety, as a pressure sensor. In particular, the potential to create a composite material characterized by extraordinary sensitivity has been illustrated through the application of microstructured conductive coatings onto woven fabrics.^[^ ^23^, ^24^ ^]^ Initially, these conductive fabrics exhibit relatively low electrical conductivity due to insufficient contact among the individual conductive fibers. Despite this limitation, they embody a promising avenue for the development of high‐sensitivity pressure sensors. The inherent porosity of these fabrics provides considerable scope for deformation in response to external pressures, and subsequent alterations in the contact areas between fibers, consequent to physical deformation, can engender a marked increase in electrical conductivity.^[^ ^25^ ^]^

The selective detection of physical deformation attributable solely to pressure remains a paramount consideration. Particularly, stretchable piezoresistive pressure sensors, which exhibit a pronounced sensitivity to external strains, necessitate the attainment of strain immunity characteristics for their viable commercialization. Instances exist wherein stretchable pressure sensors, impervious to strain, have been developed; however, these have invariably been predicated on capacitive or triboelectric modalities.^[^ ^26^, ^27^, ^28^, ^29^ ^]^ To our knowledge, there has yet to be reported the development of piezoresistive pressure sensors demonstrating strain insensitivity. This is largely attributed to the propensity of the electrical resistance within the conductive layer to undergo more significant alterations in response to strain as opposed to pressure.^[^ ^30^, ^31^ ^]^ Consequently, the imperative for piezoresistive pressure sensors to possess strain immunity is underscored. This paradox highlights a notable technical challenge; despite the critical need for strain immunity, the path to achieving this remains elusive, necessitating the innovation of new technological solutions. Furthermore, it is regrettable to note that existing studies on capacitive‐type or triboelectric‐based pressure sensors also manifest a conspicuous absence of a thorough evaluation regarding the strain‐insensitive properties across the entirety of the array system. This lacuna underscores a broader imperative for comprehensive methodological advancements that not only address the specificities of sensor sensitivity but also ensure the holistic resilience of the sensor array to external mechanical influences.

Within this study, we delineate our investigation into the fabrication of a sensor array characterized by its resistance to strain, facilitated by the development of highly sensitive pressure sensors. Our methodology involved synthesizing and electrospinning polybutadiene‐urethane (PBU), a polymer capable of reversible cross‐linking, followed by the application of a MXene nanosheet coating to create a conductive fabric. To enhance sensitivity, we strategically reduced the initial current value. The unique property of limited contact between MXene‐coated PBU fibers in the absence of pressure enabled the achievement of low initial current values, crucial for heightened sensor sensitivity. Furthermore, we pursued exceptional sensitivity by integrating semi‐cylindrical electrodes, fabricated from PBU fiber with Ag nanowires (AgNWs) selectively coated on the convex surfaces for optimal electrical conductivity. To mitigate electrode layer damage due to stretching, AgNW coating was applied under 30% strain, resulting in a corrugated configuration. These electrodes, positioned above and below the sensor, initially engage a minimal contact area due to their semi‐cylindrical shape, thereby expanding the conduction path and amplifying the electrical signal in response to pressure. The synthesized PBU, exhibiting reversible cross‐linking properties under mild temperatures, was employed to enhance the adhesive strength between the electrode and the sensor's conductive layer. PBU also served as both the substrate and encapsulation layer, safeguarding the device. The electrode's flat surface, devoid of AgNW coating, ensures direct contact with the PBU substrate and encapsulation layer, excluding the interfaces between the electrode's AgNW layer and the sensor. Through thermal control of cross‐linking, we achieved a tightly packed interface, leveraging PBU's reversible cross‐linking capabilities for improved device integrity.

Herein, we report the fabrication of a 10 by 10 sensor array, comprising 100 pressure sensor pixels, achieving an exceptional sensitivity of up to 888.79 kPa^−1^ through meticulous design of the sensor and electrode structure. Moreover, the application of a pre‐strain method in electrode construction rendered the entire array system impervious to strain, maintaining electrical properties akin to its initial state even upon expansion. To address the challenge of sensitivity variation across different pressure ranges—a common issue in soft material‐based pressure sensors—we employed artificial intelligence (AI). Specifically, we utilized a 1D convolutional neural network (1D CNN) to mitigate the material limitations, processing current fluctuation data relative to applied pressure. The model exhibited a high test accuracy of 94.08%, illustrating the potential to enhance pressure sensor performance by enabling precise pressure detection within lower sensitivity ranges. Our endeavors culminate in the advancement of a device development strategy that significantly elevates the reliability and efficacy of stretchable piezoresistive pressure sensors, addressing critical limitations in current sensor technology.

## 2 Results and Discussion

**Figure 1:**
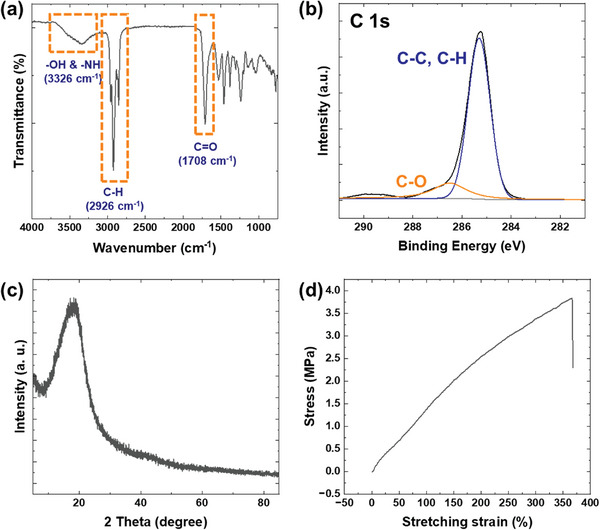
Characterization of the chemical and mechanical properties of synthesized PBU film. Panel a) displays the FT‐IR spectroscopy results, illustrating the vibrational signatures of functional groups within the PBU film. Panel b) depicts the C 1s XPS spectra, revealing the elemental composition and chemical states of carbon in the PBU film. Panel c) presents the XRD pattern of the PBU film and panel d) shows the stress‐strain curve obtained from tensile testing of the PBU film, highlighting its mechanical resilience and elasticity.

PBU, a polymer designated for the manufacture of pressure sensors, was synthesized. Within PBU, the polyurethane backbone integrates with the furan ring, as depicted in Scheme S1 (Supporting Information), facilitating a chemical reaction between these furan rings and maleimide groups to form Diels‐Alder (DA) adducts at a temperature of 60 °C.^[^ ^32^ ^]^ The resultant DA adducts embedded in the PBU matrix are capable of undergoing a retro‐DA reaction at 120 °C, which reverses the initial cross‐linking. This reversal subsequently permits the re‐initiation of the cross‐linking process at 60 °C. A schematic representation illustrating the reversible mechanism of the DA and retro‐DA reactions within PBU is provided in Scheme S2 (Supporting Information). The chemical and mechanical attributes of the synthesized PBU are documented in **Figure** **1**. The Fourier‐transform infrared (FTIR) spectrum identified three distinct peaks at 1708, 2926, and 3326 cm^−1^, corresponding to the vibrations of C═O, C─H, and ─OH and ─NH functional groups, respectively (Figure 1a). Deconvolution of the C 1s X‐ray photoelectron spectroscopy (XPS) spectrum revealed peaks at 285.0 and 286.5 eV, attributed to C─C and C─H, and C─O bonds, respectively (Figure 1b). The congruence of the XPS and FTIR data corroborates the successful synthesis of the polyurethane‐based PBU.^[^ ^33^ ^]^ The X‐ray diffraction (XRD) pattern, shown in Figure 1c, exhibited a singular diffraction peak at ≈20°, a characteristic consistent with other urethane‐based polymers.^[^ ^34^ ^]^ The evaluation of the PBU film's mechanical strength through tensile testing is depicted in Figure 1d, where the film demonstrated elongation at break exceeding 350%, indicative of PBU's inherent elasticity. The measured tensile strength of PBU, ≈15 MPa, aligns with that of comparable polyurethane‐based polymers.^[^ ^35^ ^]^ Further elucidation of this behavior is presented in Figure S1 (Supporting Information), which displays a hysteresis loop during periodic tensile testing at 100% strain. Notably, successive cycles exhibit a decrease in the area of this hysteresis loop, indicative of the viscoelastic properties of PBU. This reduction in loop area signifies the material's recovery capabilities and its ability to adapt to repeated mechanical stress through molecular realignment and optimization within its polymeric matrix. Moreover, the observed decrease in hysteresis loop area over sequential cycles suggests an ongoing molecular adaptation within the polymer structure. This adaptation involves the reconfiguration of long‐chain molecular bonds, optimizing the network's mechanical efficiency and its capacity to dissipate energy. Such molecular rearrangement, commonly referred to as “molecular training”, enhances the material's structural alignment and resilience. This dynamic restructuring is integral to the performance of stretchable polymers, enabling them to maintain functional integrity under repeated and significant deformation. This comprehensive analysis not only affirms PBU's suitability for demanding applications but also underscores its robust mechanical properties, making it an exemplary candidate for uses necessitating durable and flexible materials.^[^ ^36^ ^]^ These mechanical properties affirm the suitability of PBU for application in stretchable electronic devices, as evidenced by the assessments of elongation at break and tensile strength.

**Figure 2:**
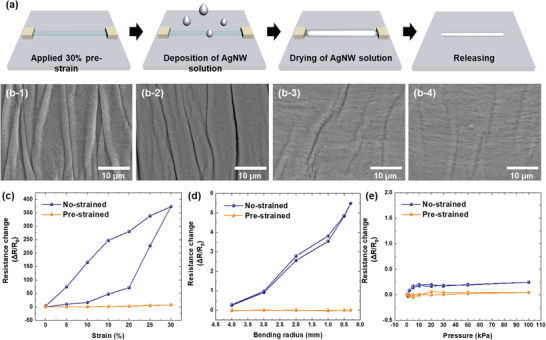
Fabrication and characterization of pre‐strained AgNWs/PBU electrodes. Panel a) depicts a schematic representation detailing the step‐by‐step process for creating pre‐strained AgNWs/PBU electrodes. Panel b) showcases FESEM images of the electrodes subjected to various levels of post‐strain (0%, 10%, 20%, and 30%), illustrating the morphological changes. Panels c–e) present comparative analyses of resistance variations in non‐strained versus pre‐strained electrodes under diverse conditions: c) strain ranging from 5% to 30%, d) bending radii from 0.3 to 4 mm, and e) applied pressures from 1 to 100 kPa, highlighting the electrodes' mechanical and electrical resilience.

Prior to the construction of the pressure sensor, the development of conductive fibers was undertaken to serve as stretchable electrodes. **Figure** **2**a provides a visual sequence of the fabrication process for AgNWs/PBU‐based electrodes. Through electrospinning, singular PBU fibers of consistent diameter were produced, adopting a semi‐cylindrical shape where only the upper side was convex, attributed to their placement on a level surface during formation. Subsequently, a coating of AgNWs was applied to the surface of these electrospun PBU fibers to generate conductive fibers. Essential for their application as stretchable electrodes, it was imperative that these fibers maintain their electrical properties under mechanical deformation. To this end, a pre‐strain technique was employed to ensure the electrodes' electrical characteristics were preserved post‐stretching. The PBU fiber was secured under a condition of 30% applied strain, onto which an AgNW solution was deposited. This fiber was then affixed to a PDMS substrate, which had undergone a hydrophilic modification via plasma treatment. The unique interfacial properties between the O_2_ plasma‐treated PBU and the hydrophobic PDMS resulted in the AgNWs, dispersed in DI water, preferentially adhering to the PBU side, achieving a uniform coating.^[^ ^37^ ^]^ The higher the pre‐strain applied, the greater the mechanical deformation the electrode can endure. However, it is crucial to ensure that this process is conducted without inflicting damage on the electrode. To determine the optimal maximum strain application condition, a series of experiments were conducted with an AgNW solution applied to PBU fibers under varying length deformations ranging from 10% to 40%. The digital images of the electrodes, post strain removal and after a relaxation period of 12 h, are presented in Figure S2 (Supporting Information). All PBU fibers utilized in these experiments were standardized to a length of 30 mm. Observations were made from both the top and side perspectives following the relaxation of the electrodes coated with the AgNW layer. Notably, only the sample subjected to a 40% pre‐strain did not revert to its original length. When observed from the side, this sample exhibited a tendency to roll toward the side devoid of the AgNW coating. This behavior is attributed to the differential capacity for strain recovery between the PBU fibers and the AgNW layer. While the PBU fibers can effectively accommodate a 40% strain, the AgNW layer cannot withstand the resultant compressive stress during relaxation. If the bent electrode is forcibly unfolded, it introduces unexpected stress to the electrode layer, thereby increasing the likelihood of cracking or peeling. These findings indicate that the maximum pre‐strain that can be applied to the electrodes, without compromising their structural integrity, is 30%.

In optimizing the performance of our pressure sensors, significant importance is placed on the electrode's ability to transmit electrical signals efficiently without loss. This ensures that the sensor's output reflects accurate pressure measurements under varying conditions. To achieve this, we investigated the effect of varying the number of coatings of AgNW on the resistance properties of the electrode, with the findings illustrated in Figure S3 (Supporting Information). The results indicate a significant decrease in resistance with an increased number of AgNW coatings. Specifically, applying the coating three times was determined to be the most effective in achieving resistance levels comparable to those of standard stretchable electrodes used in contemporary sensory applications. This triply coated AgNW/PBU electrode configuration demonstrated an optimal balance between electrical conductivity and mechanical flexibility, registering a resistance of ≈0.8 ohms—a value that supports efficient signal transmission and enhances the sensor's responsiveness and reliability. All experimental measurements were conducted subsequent to the application of AgNW coatings on pre‐strained PBU fibers, following which the fibers were allowed to relax. This process simulates real‐world operational conditions where the fibers are subjected to various degrees of mechanical stress and relaxation.

**Figure 3:**
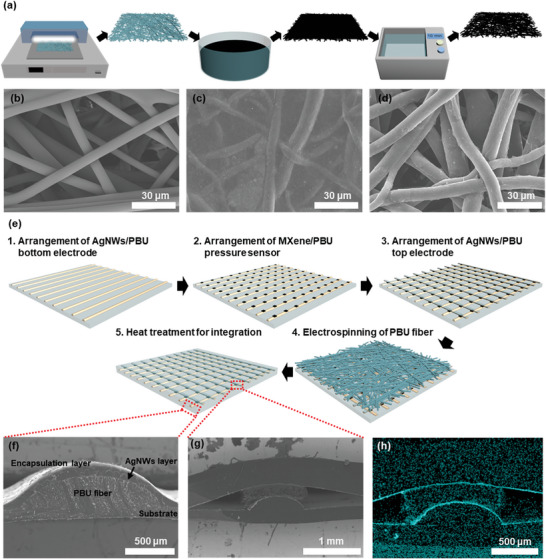
Detailed illustration and microscopic analysis of pressure sensor construction using MXene and PBU fiber matrix. Panel a) presents a schematic overview of the pressure sensor's fabrication process, highlighting the integration of MXene with the PBU fiber matrix. FESEM imagery showcasing b) the unaltered state of PBU fibers, c) MXene‐coated PBU fibers prior to ultrasonication treatment, and d) MXene‐coated PBU fibers following ultrasonication, evidencing the effect of the process on the fiber coating. Panel e) depicts the sequential assembly of the sensor array, consisting of 100 individual pixel sensors. Cross sectional FESEM views detailing f) the encapsulated AgNWs/PBU electrode structure and g) a complete unit sensor within the array. Panel h) features an EDS generated mapping image of the elemental distribution of silver (Ag) within a unit sensor, illustrating the uniformity and integration of materials.

**Figure 4:**
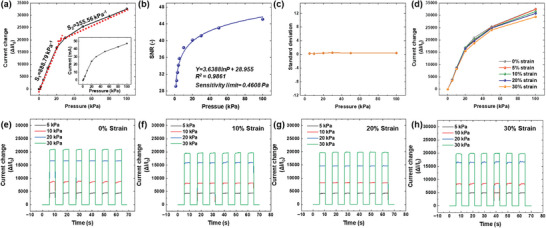
Comprehensive analysis of the MXene/PBU fiber‐based unit pressure sensor. Panel a) demonstrates the sensor's current variation relative to applied pressures spanning 1 to 100 kPa, with sensitivity evaluated within two distinct pressure intervals: P < 20 kPa and 20 < P < 100 kPa (inset shows raw plot data of current for pressure). Panel b) depicts the SNR alongside the determined sensitivity threshold. Panel c) illustrates the standard deviation in current change across varying pressures, highlighting the sensor's reproducibility. Panel d) presents the correlation between the relative current change and applied pressures under different strain conditions (ranging from 0% to 30% strain). Panels e–h) showcase the sensor's dynamic responses to cyclical pressure under stretching strains from 0% to 30%, evidencing the resilience and sensitivity of the sensor system under mechanical deformation.

Field Emission Scanning Electron Microscopy (FESEM) images (Figure 2b) document the electrode surface post‐application of strain up to 30%. The images reveal a rugged AgNW layer with visible wrinkles on the PBU fiber surface at 0% strain (Figure 2b‐1), which gradually smoothens into an almost flat appearance at 30% strain (Figure 2b‐2–4), without resulting in cracks or peeling that would impair electrical conductivity. To thoroughly investigate the complex topography of the AgNW/PBU‐based electrode structure, detailed surface scans were conducted using an atomic force microscope (AFM). The 3D scanning results, depicted in Figure S4a (Supporting Information), closely correspond to those observed in the FESEM images (Figure 2b‐1). Additionally, the height profile data presented in Figure S4b (Supporting Information) reveal that the maximum peak‐to‐valley measurement of the surface irregularities, notably the wrinkles, is ≈150 nm. Comparative analysis of resistance variations under different deformations—stretching, bending, and pressing—between pre‐strained and non‐strained AgNWs/PBU electrodes (Figure 2c–e) confirmed that the pre‐deformed electrode retained its electrical properties across all types of deformation. Conversely, the resistance of the non‐deformed electrode tended to escalate under more severe conditions, except when pressure was applied, particularly noting a resistance increase of over 350% upon 30% strain during stretching tests. To verify the durability of the pre‐strained electrode's stretchability, a series of 5000 stretching‐restoration tests were conducted with 30% strain, exhibiting stable resistance changes as depicted in Figure S5 (Supporting Information). These results demonstrate that AgNWs/PBU electrodes, engineered through pre‐deformation technology, possess the requisite mechanical stability for incorporation into stretchable electronic devices.

A detailed schematic outlining the process of fabricating the pressure sensor is presented in **Figure** **3**a. Initially, a matrix composed of PBU fibers was constructed, with fibers vertically aligned to create a randomly oriented network structure; the PBU fibers exhibited an average diameter of ≈5 µm, as depicted in Figure 3b. Subsequent to the preparation of the PBU fiber matrix, it underwent oxygen plasma treatment before being submerged in a suspension of MXene nanosheets. FESEM imagery capturing the MXene nanosheets applied is provided in Figure S6 (Supporting Information), revealing an average lateral dimension of 6.3 µm. nalysis, aimed at assessing the chemical composition of the MXene nanosheets, is illustrated in Figure S7 (Supporting Information). Within the O 1s spectrum, the emergence of peaks at 530.18 and 531.18 eV respectively denotes the presence of C‐Ti‐O_x_ and C‐Ti‐OH_x_ functional groups, indicative of the MXene nanosheets' surface chemistry. Furthermore, the peak observed at 533.1 eV, which corresponds to the presence of water molecules, highlights the inherent hydrophilic nature of MXene. This observation suggests the potential formation of stable chemical bonds between the MXene and the PBU polymer surface. Such bonding interactions are indicative of a strong affinity between the hydrophilic sites on the MXene and the functional groups on the PBU polymer, contributing to the overall stability and performance of the composite material.^[^ ^38^, ^39^ ^]^

The MXene‐coated PBU matrix subsequently underwent a heat treatment procedure to enhance the adhesion between the MXene nanosheets and the PBU fibers. During this heat treatment at 120 °C, the PBU underwent a retro‐DA reaction, gaining fluidity, followed by a DA reaction at 60 °C, facilitating the re‐cross‐linking of the PBU polymer chains. This resulted in a robust bonding to the MXene nanosheets layer enveloping the PBU fiber surfaces. Given the dip‐coating method employed for MXene application, nanosheets also infiltrated voids devoid of PBU fibers, appearing as extraneous burrs. To remove these MXene burrs, the composite was subjected to ultrasonication for 10 min. FESEM images capturing the MXene‐PBU fiber matrix pre‐ and post‐sonication are exhibited in Figure 3c,d, notably demonstrating that the MXene layer on the PBU fiber surface remained intact post‐sonication, devoid of MXene burrs. This integrity is attributed to the durable linkage between PBU fibers and the MXene layer, established through the retro‐DA and DA reactions. Energy Dispersive X‐ray Spectroscopy (EDS) mapping further elucidates the sonication's impact, with Figure S8a (Supporting Information) showcasing the dispersion of titanium (Ti), a principal element of MXene, throughout the composite, whereas post‐ultrasonic treatment, Ti's presence was predominantly localized around PBU fibers, as shown in Figure S8b (Supporting Information). To assess the interfacial adhesion between MXene and PBU within our ultrasonically treated composite matrix, we employed standardized tape tests. This method involved applying a strip of adhesive tape to the treated surface and subsequently removing it, a procedure meticulously documented in Figure S9 (Supporting Information). The results of these tests were unequivocal; the MXene layers exhibited robust adhesion to the PBU substrate, with no detachment observed following the removal of the tape. This outcome not only confirms the effectiveness of the ultrasonic treatment in enhancing the bonding strength but also demonstrates the durability of the interface under mechanical stress.

Upon the application of pressure to the engineered MXene/PBU composite, the conductive MXene/PBU fibers embedded within the matrix structure establish contact points. This contact formation consequently enhances the flow of current due to the inherent conductivity of MXene. The pressure‐induced interaction between the fibers facilitates improved electrical pathways, thereby significantly augmenting the composite's overall electrical performance.^[^ ^40^, ^41^ ^]^ This phenomenon underpins the operational principle of the pressure sensor. Figure 3e delineates a schematic representation of the process involved in constructing a 10 by 10 sensor array by the strategic assembly of AgNWs/PBU electrodes and MXene/PBU pressure sensors. This assembly was meticulously arranged vertically on the PBU film, following a sequential order from the bottom AgNWs/PBU electrodes, through the MXene/PBU sensors, to the top AgNWs/PBU electrodes. To safeguard and secure the composite components, a capsule layer was created via electrospinning. Given that the MXene/PBU sensors detect pressure through alterations in the fiber matrix's porous structure, it is imperative to prevent any changes to this structure throughout the encapsulation process. For instance, the direct application of a polymer solution for encapsulation could result in the infiltration of the solution into the matrix, potentially compromising the sensor's functionality. To circumvent these issues, an encapsulation technique employing PBU electrospinning was utilized. Since the electrospun PBU fibers are confined to the exposed surface, their application does not interfere with the pressure sensor nestled between the two electrodes. Encapsulation was thus achieved through the execution of both retro‐DA and DA reactions, culminating in the cohesive integration of the entire system.

FESEM images depicting the cross sectional view of an individual MXene/PBU sensor within the array are presented in Figure 3f,g. The semi‐cylindrical electrode, having been coated with AgNWs solely on the convex surface of the PBU fiber, results in the bottom PBU substrate film and the flat surface of the electrodes being seamlessly integrated, devoid of any discernible boundaries. The electrospun PBU fibers, in turn, amalgamate to resemble a singular film. Despite the AgNW/PBU electrode's non‐flat geometry, the PBU encapsulation layer is engineered to achieve a uniform thickness. A noteworthy aspect is the comprehensive envelopment of the PBU fibers, which constitute the pressure sensor, by MXene nanosheets possessing an extensive specific surface area. This ensures the retention of the initial porous structure post‐heat treatment, as there is no encroachment upon the inter‐fiber spaces. Moreover, the surface of the AgNW‐coated electrode is clearly demarcated from the PBU encapsulation layer, yet, due to the direct contact encapsulation of the PBU fibers with the electrode surface during electrospinning, the formation of gaps or pores is precluded, facilitating adherence to the electrode layer. Figure 3h illustrates the process of layer assembly and integration through EDS mapping, confirming the preservation of the AgNW layers in their pristine condition. SEM and EDS analyses corroborate the complete integration of the AgNWs/PBU electrodes with both the substrate and the encapsulation layer, starkly contrasting with the MXene/PBU pressure sensors which are merely positioned in contact, without physical integration between the two electrodes. Consequently, the pressure sensors maintain their independence within the system, implying that mechanical deformations impart differential stimuli on the integrated electrodes and substrate versus the pressure sensors. This attribute is critically pertinent considering the lateral expansion of the array system. Distinct from other components, the MXene/PBU pressure sensors steadfastly resist length variations and exhibit insensitivity to strain, underpinning their robust performance.

To rigorously assess the pressure‐sensing capability of the meticulously assembled unit sensor system (comprising an encapsulation PBU layer, AgNWs/PBU top electrode, MXene/PBU pressure sensor, AgNWs/PBU bottom electrode, and PBU substrate), an in‐depth investigation into the sensor's sensitivity thresholds and its performance under stretching was undertaken, with the findings comprehensively detailed in **Figure** **4**. The sensitivity metric, a quintessential factor, plays a pivotal role in determining the pressure sensor's reliability and precision. The sensitivity calculation was anchored in the equation: *S* = *δ*(*ΔI*/*I_0_*)/*δP*, wherein *ΔI* signifies the change in current resultant from applied pressure, *I_0_* represents the baseline current, and *P* denotes the exerted external pressure. Pressure‐dependent current plots are inserted inside the graph, and *I_0_* value without applied pressure was 1.45 µA. As delineated in Figure 4a, the sensor's sensitivity bifurcates into two distinct ranges: *S_1_*, within the low‐pressure realm (1 – 20 kPa), and *S_2_*, within the high‐pressure domain (20 –100 kPa). It is of paramount importance to highlight the sensor's extraordinary sensitivity, especially notable within the low‐pressure segment where it achieved a sensitivity of 888.79 kPa^−1^, a metric unparalleled in the annals of stretchable pressure sensor research. In the high‐pressure segment, a sensitivity of 355.56 kPa^−1^, which is relatively low, is recorded. This observation can be attributed to the structural characteristics of the electrode and the sensor. In the low‐pressure segment, the output current value exhibits variation due to the changing contact area between the upper and lower electrodes and the pressure sensor. Additionally, the degree of contact between the conductive fibers within the pressure sensor fluctuates, thereby increasing the number of conductive pathways. This results in a doubling of the current amplification effect in response to the increased pressure. Conversely, in the high‐pressure segment, the current amplification effect is relatively minimal. This is because the increase in the contact area between the electrode and the sensor, or the formation of conductive pathways within the pressure sensor, has already been significantly realized. Nevertheless, the observed improvement in current with increasing pressure is due to the pressure sensor's composition of PBU fibers, which possess excellent elasticity. In the low‐pressure region, contact between the conductive fibers within the pressure sensor occurs predominantly when the fibers are in close proximity. However, under higher pressure, the MXene/PBU fibers undergo greater deformation, which facilitates contact even between fibers that are relatively distant. This enhanced contact potential leads to a steady improvement in current value as pressure increases. Despite this, sensitivity decreases because the pronounced current amplification effect observed in the low‐pressure region is diminished. However, it is important to note that the reduced sensitivity still remains at a superior level.

The remarkable sensitivity observed in the pressure sensor is largely due to its meticulously engineered structural design. Central to the sensor's architecture are the PBU fibers, methodically arrayed in sequence via electrospinning, subsequently enveloped in a conductive layer of MXene. This configuration, particularly when viewed in cross section, facilitates the formation of a limited number of conductive pathways, localized primarily at junctures where the cylindrical fibers intersect. Furthermore, the sensor establishes connections with AgNWs/PBU‐based electrodes at both the top and bottom, crucial for the relay of electrical signals engendered by the sensor. The electrodes themselves are characterized by a semi‐cylindrical morphology, with only the convex portions making initial contact with the sensor. This results in a relatively low initial current value (*I_0_*), attributable to the inherent design of both the electrode and sensor. It is discernible from the sensitivity formula that a diminished *I_0_* is beneficial, serving to amplify the sensor's sensitivity. Another salient feature contributing to the enhanced sensitivity is the dynamic interaction that ensues upon the application of force. Not only does the applied force augment the number of contact points between the conductive MXene/PBU fibers within the sensor, but it also induces deformation in the stretchable electrodes, thereby altering the contact area with the sensor. Such modifications significantly elevate the *ΔI*/*I_0_* ratio, catalyzing the observed increase in sensitivity. This synergy between structural ingenuity and functional adaptability underpins the exceptional performance of the pressure sensor, evidencing the critical role of design in achieving high sensitivity.

To elucidate the impact of the initial contact area between the electrodes and the pressure sensor on its sensitivity, an experiment incorporating a flat electrode was undertaken, with the resultant sensitivity assessed and depicted in Figure S10 (Supporting Information). Upon application of a 20 kPa pressure, the *S_1_* and *S_2_* were discerned to be 40.82 and 8.90 kPa^−1^, respectively. These figures are markedly inferior to those obtained using a semi‐cylindrical electrode, unequivocally demonstrating that the sensitivity enhancement was facilitated by the strategic modification of the electrode's geometric form. An in‐depth analysis of the sensor's theoretical sensitivity threshold was conducted using polynomial fitting techniques. This analysis specifically utilized the signal‐to‐noise ratio (SNR) as a critical parameter, which is significantly affected by variations in the externally applied pressure. The relationship between SNR and applied pressure was methodically quantified to determine the sensor's response characteristics under varying conditions. These findings are comprehensively illustrated in Figure 4b, where the polynomial fitting curve provides a visual representation of how the SNR varies with changes in pressure, thus offering insights into the sensor's dynamic range and its capability to detect subtle variations in environmental pressure. The SNR was quantified as 10 × log_10_(*P_signal_*/*P_noise_*), wherein *P_signal_* and *P_noise_* represent the signal and noise amplitudes, respectively. The polynomial fitting yielded an equation of *Y* = 3.6388 ln*P* + 28.955, accompanied by a fit evaluation index (*R^2^*) of 0.9861, where *P* denotes the applied pressure and *Y* the SNR. The *R^2^* value serves as a testament to the alignment between the empirical data derived from the sensor and the theoretical fitting curve, with a value approaching 1 corroborating the data's veracity. The sensitivity threshold, typically defined by the SNR value of 1, was astonishingly low at 0.4608 Pa for the sensor in question. This revelation posits that our sensors possess the potential capability to discern exceedingly minute pressures, highlighting the profound sensitivity achievable through meticulous design and calibration of the sensor system.

Reproducibility, defined as the uniformity of current output changes upon repeated application of identical pressure to a sensor, was meticulously evaluated through a rigorously controlled experiment involving ten fabricated samples. For each specimen, eight distinct pressures were applied tenfold, with the ensuing changes in current meticulously documented. The standard deviation of these observed changes was subsequently plotted to serve as a metric for evaluating reproducibility (Figure 4c). The recorded standard deviation exhibited a marginal increment with escalating applied pressures, yet such variations were deemed negligible, underscoring the consistent performance and reliability of both the sensor's properties and its fabrication methodology. The uniformity afforded by the electrospinning process facilitates the creation of a matrix comprising fibers with consistent diameters across a broad surface area. This uniformity is instrumental in achieving high reproducibility in the fabrication of pressure sensors. Consequently, the results from this fabrication method indicate that the signal output from the assembled sensor array is exceptionally reliable. The inherent precision of the electrospinning technique thus ensures the structural and functional integrity of the sensor system. Another critical attribute of the pressure sensor is its response time, encompassing both the ascent and descent times across a pressure spectrum of 5 to 30 kPa, as delineated in Figure S11 (Supporting Information). The impact of pressure magnitude on response velocity was found to be minimal, with all measured response times exceptionally swift, clocking in at less than 0.6 ms. This rapidity is attributed to the instantaneous physical deformation upon the application and removal of pressure, facilitated by the superior elasticity of the PBUs constituting the bulk of the device's material. Figure 4d presents findings related to the sensor's pressure‐sensing capabilities under various uniaxial strains. Notably, even with the imposition of a 30% strain, the sensor's performance did not significantly deteriorate; however, a slight reduction in the rate of current change was observed as the level of strain increased. This suggests that the sensor maintains its functional integrity under strain, albeit with minor modifications in its sensitivity to pressure changes.

Figure S12 (Supporting Information) elucidates the findings from an analogous experiment executed exclusively with pre‐strained AgNWs/PBU electrodes, devoid of an integrated pressure sensor. Upon the application of strain under varied conditions, the recorded current change remained substantially uniform, suggesting that the alterations in electrode properties exert minimal influence on the experimental outcomes. Notably, even when subjected to a strain of 30%, the electrodes demonstrated a robust insensitivity, a phenomenon that can be ascribed to the application of fine compressive stress in a vertical orientation during the device's horizontal elongation. The rationale behind the minimal impact on current change, despite considerable strain, lies in the structural composition of the device. Specifically, the region encompassing both electrodes and the sensor represents the thickest segment of the device, thereby enduring comparatively less stress than its thinner counterparts under uniform strain. Given that MXene is an inorganic material lacking elasticity, it is inherently susceptible to strain. Nonetheless, within the encapsulated assembly, only the PBU substrate, the PBU encapsulation layer, and the two AgNWs/PBU electrodes are cohesively integrated via the DA reaction, leaving the MXene/PBU pressure sensor mechanically detached and thus shielded from strain. Figure 4e–h delineate the dynamic responses to cyclical pressure stimuli on the sensors under strain, showcasing the system's capacity to discern pressure with exceptional sensitivity. The graphs of current change were calculated from the current‐pressure curve shown in Figure S13 (Supporting Information). The initial current measurements recorded in the absence of applied pressure, but under varying degrees of strain, were 1.67, 1.74, 1.88, and 1.93 respectively. These values were obtained at incremental strain levels, illustrating that despite the mechanical deformation, the electrical performance of the sensor system remained stable. This stability in current readings across different strains demonstrates the robustness of the electrode and sensor materials, confirming that their intrinsic electrical properties are not compromised even when subjected to mechanical stress. To rigorously assess the durability of our newly developed sensor system, we conducted a comprehensive pressing‐releasing durability test. This test involved subjecting the sensor to a consistent pressure of 30 kPa over a series of 10000 cycles, during which the changes in electrical current were meticulously recorded, as depicted in Figure S14 (Supporting Information). The stability of the current response under pressure was observed to be remarkably consistent throughout the testing process, with minimal evidence of signal degradation. This sustained performance can be attributed primarily to the robust encapsulation of the sensor components. The sensor system and its electrodes are effectively sealed within a protective layer, ensuring optimal isolation and mechanical stability. Furthermore, the integration of the active materials, MXene and AgNW, with the PBU matrix, has been optimized to achieve excellent mechanical coupling. These results affirm the robustness of the sensor assembly, comprised of the engineered MXene/PBU pressure sensor, dual AgNWs/PBU electrodes, alongside a PBU substrate and encapsulation layer, in delivering reliable and high‐fidelity pressure detection.

Pressure sensors attain utility only when their reliability, precision, and operational excellence are unequivocally established. Moreover, their applicability is confined to singular sensor implementations unless they are fabricated into arrays comprising multiple sensing units. The performance of pressure sensors fabricated in this research was rigorously assessed across various metrics, including sensitivity and response speed. For an objective evaluation of these indices, the attributes of the sensors were benchmarked against those of recently developed flexible/stretchable pressure sensors, with the comparative analysis detailed in **Table** **1**.^[^ ^41^, ^42^, ^43^, ^44^, ^45^, ^46^, ^47^ ^]^ This comparison highlighted that the response time, detection threshold, and operational range of the pressure sensor delineated in this study are competitive with, or surpass, those of existing counterparts. A critical observation emerged from this analysis: nearly all sensor arrays have traditionally employed flat flexible films for electrodes, indicating a lack of prior endeavors to enhance sensitivity through electrode shape modification. The pronounced sensitivity achieved by our pressure sensing system, which incorporates a semi‐cylindrical electrode, underscores the efficacy of electrode shape transformation as a strategy for sensitivity augmentation. Furthermore, despite the mechanical adaptability offered by piezoresistive‐type pressure sensors, there has been an absence of consideration for strain‐induced effects within this context. To our knowledge, no piezoresistive pressure sensor exhibiting strain immunity has been documented to date, attesting to the innovative nature of our developed sensor array. While the performance analysis table does cite a pressure sensor with superior sensitivity to the one discussed herein, the critical aspect of strain immunity significantly influences practical applicability. Hence, the strain‐immune pressure sensor developed through this research stands on the cusp of commercialization, distinguished by its advanced operational capabilities.

**Table 1:** Performance comparison of recent flexible/stretchable piezoresistive pressure sensor arrays. This table presents a detailed comparative analysis of key performance parameters across a selection of recently documented piezoresistive pressure sensor arrays, highlighting advancements and innovations in the field.

Approach	Sensitivity	Sensing range	Detection limit	Rise/relaxation time	Strain immunity	References
Semi‐cylinder AgNW/PBU electrode/MXene‐PBU fibrous sensor/ Semi‐cylinder AgNW/PBU electrode	888.79 kPa^−1^ (< 20 kPa) 355.56 kPa^−1^ (20–100 kPa)	< 100 kPa	0.4608 Pa	66 / 69 ms	Yes	This work
PS‐MXene‐GO sponge sensor/ Ag‐PI electrode	115 kPa^−1^ (< 7.58 kPa) 224 kPa^−1^ (7.58–20.65 kPa)	< 20.65 kPa	–	63 / 40 ms	No	[41]
Vertically aligned Ni‐coated Carbonfiber/ Cu‐Kapton electrode	228 537 kPa^−1^ (< 20 kPa) 15 525 kPa^−1^ (20‐100 kPa)	< 100 kPa	–	30 / 31 ms	No	[42]
PDMS‐Ti‐Au microdome/ LIG electrode	3.1 kPa^−1^ (1 Pa–13 kPa) 0.22 kPa^−1^ (13–400 kPa)	<400 kPa	1 Pa	14.93 / 22.41 ms	No	[43]
Cu‐Au‐PI electrode/ Graphene‐PDMS foam/ Cu‐Au‐PI electrode	50.45 kPa^−1^ (< 50 Pa) 4.35 kPa^−1^ (50–400 Pa)	< 400 Pa	0.209 Pa	39 ms / –	No	[44]
Graphene‐PDMS foam/ Cr‐Au‐PI electrode/	1.37 kPa^−1^ (< 80 kPa)	< 80 kPa	–	20 ms / –	No	[45]
MXene paper/ Ag‐Ni‐PI electrode	28.43 kPa^−1^ (< 1.9 kPa) 6.31 kPa^−1^ (1.9–4.2 kPa) 1.62 kPa^−1^ (4.2–7 kPa)	< 7 kPa	0.8 Pa	98.3 / 99.3 ms	No	[46]

**Figure 5:**
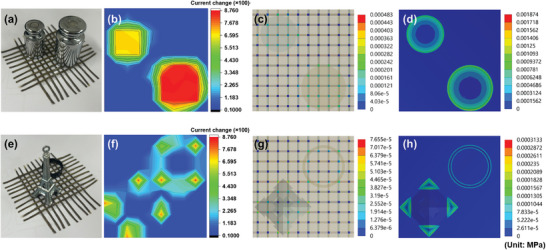
Evaluating the response characteristics of arrayed sensors to pressure exerted by diverse objects. Panels a) and e) display digital photographs showcasing the sensor arrays under examination. Panels b) and f) illustrate distribution charts mapping the current change across individual sensor pixels. Panels c) and g) present the stress distribution exerted on the MXene/PBU‐based pressure sensor. Panels d) and h) depict the stress distribution within the encapsulation layer. Panels a–d) examine the response to a weighted object, while panels e–h) investigate the effects of placing a model of the Eiffel Tower and a ring on the sensor array, respectively, demonstrating the sensor's ability to distinguish between varying pressure stimuli.

Ultimately, the efficacy of pressure sensing was evaluated through the deployment of various objects atop an array encompassing 100 pressure sensor pixels, to ascertain the system's practical applicability. Objects of diverse shapes and masses were strategically placed at random coordinates across the array, as depicted in **Figure** **5**a,e. Following this, the instantaneous current variation for each sensor pixel was scrupulously recorded (Figure 5b,f). Pixels spanning the full area under the influence of weight exhibited the most pronounced current shifts, with a radial gradient observable from pixels subjected to pressure solely in specific regions of the sensor. In this context, the applied weights exerted pressures of 1.5 and 2 kPa, respectively. This experiment aimed to evaluate the sensor array's proficiency in discerning pressure differentials elicited by distinct objects, with the findings showcased in Figure 5e–h. The experiment entailed positioning both an Eiffel Tower replica and a ring on the sensor array, capturing the rate of current change instantaneously. The Eiffel Tower replica, featuring four triangular legs as illustrated in Figure S15 (Supporting Information), manifested the largest current shift when the object fully enveloped the sensor's surface area. Simulated stress distribution on the sensors is explicated in Figure 5c,d,g,h, complemented by technical schematic delineating component dimensions in Figure S16 (Supporting Information). Figure 5c,g elucidates the stress distribution diagrams for the MXene/PBU pressure sensors, revealing stress concentration due to surface elastic deformation resulting from object placement. This pattern mirrors the current change distribution depicted in Figure 5b,f. Conversely, Figure 5d,h delineates stress distribution for the PBU encapsulation layer, highlighting a comparatively lower stress intensity at the contact area's center compared to its periphery. These simulation outcomes infer that sensor responsiveness is predominantly influenced by alterations in the contact area rather than by stress‐strain modifications. Consequently, MXene/PBU‐based pressure sensors exhibit pronounced sensitivity in pressure detection, affirming their efficacy and potential for diverse applications. The spatial resolution of a sensor array is intrinsically linked to the density of its sensors within a given area. Higher sensor density typically enhances the array's capability to discern and map spatial variations more precisely. To rigorously test this relationship, we assessed the capacity of individual sensors within the array to accurately detect and respond to applied pressures independently of one another, particularly focusing on their performance in conditions of varying sensor spacing. As depicted in Figure S17 (Supporting Information), we conducted experiments where a uniform pressure of 1 kPa was applied directly to the central sensor within setups where the spacing varied from 1 to 4 mm. Remarkably, even at the closest interval of 1 mm, the system displayed robust isolation between sensors, with no observable crosstalk affecting the detection capabilities. This effective isolation is attributable to the unique properties of the PBU used in constructing the surrounding layers of each sensor. The excellent elasticity of PBU ensures that the mechanical strain induced by pressure application is contained within the immediate vicinity of the sensor being activated, thereby preventing any significant mechanical influence on adjacent sensors. This experimental setup not only confirms the individual sensors' ability to operate independently without interference but also validates the practical scalability of reducing sensor spacing to enhance spatial resolution. The implications of these findings are substantial for applications requiring high‐resolution tactile feedback, as they demonstrate the potential to significantly increase sensor array density without compromising the accuracy and reliability of the data collected. These results support the development of more compact and efficient sensor arrays that can be effectively utilized in advanced robotics, medical diagnostics, and interactive technology.

**Figure 6:**
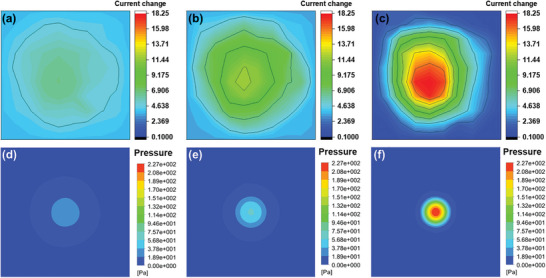
Wind pressure measurement analysis. This figure illustrates the sensor array's response to wind pressure applied at varying distances. Panels a–c) depict graphs of current changes when wind of uniform intensity is directed toward the sensor array from distances of 100, 50, and 20 mm, respectively, highlighting the sensitivity of the sensor to spatial variations in wind pressure. Panels d–f) present CFD simulations that map the pressure distribution across the sensor surface when subjected to wind emitted from the aforementioned distances, visually demonstrating the impact of proximity on pressure dispersion and intensity.

The fabricated pressure sensor showcases unparalleled sensitivity, capable of discerning subtle variations, including fluid flow and direct pressure alterations ensuing from the immediate positioning of objects. Moreover, its configuration as an array significantly broadens its pressure detection capabilities. To illustrate this, an experiment was conducted where a wind speed of 3 m s^−1^ was directed at a 10 by 10 sensor array using an air gun, with the resultant electrical signals from each sensor pixel meticulously recorded. During the trial, the air gun was stationed at distances of 100, 50, and 20 mm from the array. The nozzle of the air gun measured 7 mm in diameter, with the airflow targeted at the array's center. Despite the air being expelled from a nozzle of constant diameter, the dispersion and distribution of wind energy were observed to increase with distance, enhancing the pattern of wind dispersion. In contrast, air released at closer proximity to the array manifested minimal energy dispersion, concentrating the impact at a specific locale. This dynamic is accurately depicted in the current change distribution (**Figure** **6**a–c). Figure 6d–f depicts simulated pressure distribution on the sensor surface, derived through computational fluid dynamics (CFD), which vividly illustrates the variance in wind energy dispersion relative to distance. Predominantly, the simulation reveals that the central region of the sensor array bears the brunt of the pressure, which diminishes radially toward the periphery. Additionally, a more confined pressurized region emerges at closer proximities to the air source. These CFD findings strongly align with the observed changes in current, underscoring the sensor's exquisite sensitivity. Notably, only a sensor of such high sensitivity could detect the wind speed of 3 m s^−1^ employed in this experiment—a velocity so gentle it merely causes leaves to flutter. Moreover, discerning the presence and fluctuations of an invisible medium like wind poses a substantial challenge, particularly when relying on the output from a solitary sensor, which may not unequivocally indicate changes in wind intensity or distance from the source. However, by leveraging an array comprising 100 highly sensitive sensors, the origins of current variations were distinctly ascertainable, affirming the array's efficacy in accurately capturing and interpreting subtle environmental changes.

The developed pressure sensor exhibits exceptional sensitivity, enabling the detection of minute variations, such as fluid flow and direct pressure changes upon the direct placement of the object. In addition, since it is manufactured as an array, the ability to detect pressure can be extended extensively. To demonstrate this, a wind speed of 3 m s^−1^ was fired at a 10 × 10 sensor array with an air gun, and electrical signals generated from each sensor pixel were detected. During the experiment, the air gun was positioned at distances of 10, 5, and 2 cm from the sensor array. The air gun's pipe diameter was 0.7 cm, and the air was released toward the center of the sample. Even if air is emitted from a source with the same diameter, the further the distance, the more distributed and scattered the wind energy, increasing the wind dispersion pattern. Conversely, if a wind of the same speed is emitted at a closer distance, relatively little dispersion of energy occurs and is concentrated at a specific location. This principle is well reflected in the distribution of current changes (Figure 6a–c). Figure 6d–f is a simulated pressure distribution diagram of the surface calculated through computational fluid dynamics (CFD), which also effectively shows the difference in wind energy dispersion with distance. Overall, it indicates that the center of the sensor array experiences the highest pressure, and the pressure applied decreases radially as it is located on the outer edge. Furthermore, a smaller pressurized area is observed at a shorter distance from the source. These CFD results show a strong correlation with changes in current. In this part of our study, we conducted a detailed mapping of current distribution across the sensor array under controlled conditions where the angle of wind irradiation was systematically varied. This experiment was conducted with consistent spacing between the wind source and the sensor array and at a uniform wind speed, as depicted in Figure S18 (Supporting Information). The apparatus utilized, an air gun, was precisely positioned 20 mm from the center of the array and oriented to emit wind at a 45‐degree angle toward the north and west, relative to the vertical axis. The directional application of the wind toward the center of the array was critical in our analysis. It was observed that the central location within the array registered the highest variation in the current response. This measurement notably decreased in a gradient fashion as the distance from the center increased, aligning with the directional flow of the wind. These findings are significant as they demonstrate the capability of the pressure sensor array not merely to measure the intensity of the wind but to accurately discern the direction from which the wind is emanating. Such directional sensitivity is essential for applications requiring precise environmental monitoring and navigation assistance, where understanding both the force and the flow direction of wind is crucial. Only a high‐sensitivity pressure sensor can detect the wind speed of 3 m s^−1^ fired in this experiment because the wind speed is so weak that the leaves shake lightly. Even more, it is difficult to prove the existence of an invisible flow of fluid such as wind. Therefore, it is difficult to determine whether the wind strength has changed or is farther from the source from the current value output from a single sensor. However, because the manufactured pressure sensing element implemented an array consisting of 100 sensors with high sensitivity, the cause of the current change could be clearly identified.

**Figure 7:**
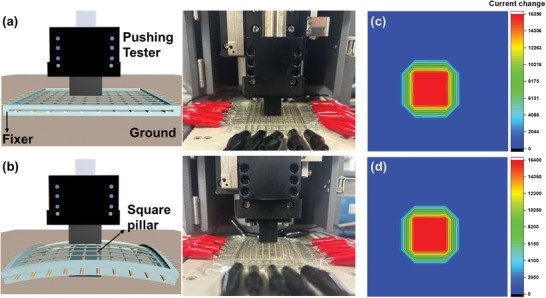
Illustrating strain immunity in arrayed pressure sensors. This figure presents schematic depictions alongside actual images of the sensor array a) in its unaltered, planar form and b) under applied strain. Panels c,d) showcase color‐coded contour maps depicting the variation in current across the array corresponding to the conditions in (a) and (b), respectively, effectively visualizing the sensor array's remarkable ability to maintain accurate pressure sensing despite deformation.

A pressure response system, impervious to strain, has been engineered employing pre‐deformed electrodes and meticulously calibrated sensor configurations. To validate its efficacy, experiments depicted in **Figure** **7**a,b were executed. Within these trials, a square pillar was utilized to exert a direct pressure of 10 kPa upon the central nine sensor pixels. The variations in current output for all 100 pixels, inclusive of the nine subjected to pressure, were documented both in the array's unaltered state and upon its physical deformation. This deformation at the array's core was facilitated by an additional square pillar, mirroring the push tester's square pillar in surface area. The sensor array was anchored along its periphery with rigid glass to ensure a uniform deformation across the assembly. In the conducted experiment where identical deformation was applied to an unstrained AgNW/PBU electrode, we observed a significant alteration in the electrical properties of the device. Specifically, the resistance of the electrode increased by ≈350%. This substantial change underscores the electrode's sensitivity to mechanical stress. Furthermore, using the calibrated analysis depicted in Figure 2c, the strain induced by the square pillar on the sensor array was quantitatively assessed to be ≈25%. This estimation is critical as it allows for a precise correlation between mechanical strain and electrical response, providing valuable insights into the material's behavior under applied stress. Such data are essential for the validation of the electrode's performance in practical applications, where flexibility and durability under mechanical deformation are required. The resultant current change values are visually represented through contour graphs in Figure 7c,d, which astonishingly revealed a striking similarity in outcomes under both conditions, rendering them nearly indistinguishable. This outcome emanates from the synergistic integration of three fabrication technologies: 1) The utilization of pre‐strained AgNWs/PBU electrodes, 2) The strategic placement of MXene/PBU pressure sensors to function autonomously without any physical junctions, and 3) The implementation of an effective strain distribution strategy through differential thickness across areas hosting the pressure sensor versus other regions. Upon the application of external strain to the entire system, the stress predominantly disperses across the thinner segments, where solely the PBU film is present. Although some strain impacts the AgNW/PBU electrodes, which are physically integrated with either the PBU substrate or the encapsulation layer, their pre‐strain fabrication ensures their resilience. The MXene/PBU sensors, being unattached, circumvent external strain influences, thereby retaining their pre‐deformation pressure detection capabilities. This distinctive attribute of the developed sensor array, stemming from an innovative structural design encompassing electrodes, sensors, substrates, and encapsulation layers, heralds a pioneering advancement in the domain of high‐sensitivity piezoresistive pressure sensors. It introduces a novel paradigm for the creation of stretchable pressure sensors that merge heightened sensitivity with inherent strain immunity, marking a significant leap forward in sensor technology.

**Figure 8:**
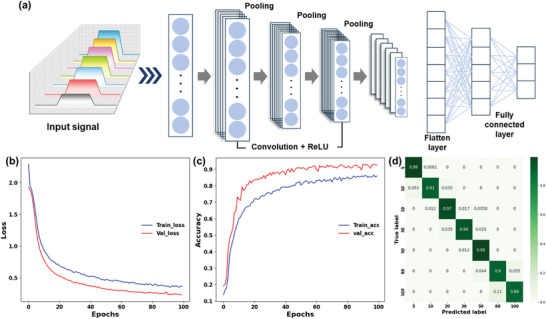
Deep learning integration and performance analysis of the 1D‐CNN model for pressure sensing. Panel a) provides a schematic representation of the 1D‐CNN architecture utilized. Graphical depictions illustrating the evolution of b) training loss and c) training accuracy with an incremental number of epochs are presented, demonstrating the model's learning efficiency. Panel d) displays the confusion matrix for the CNN model, highlighting its predictive accuracy across various conditions.

Upon meticulous evaluation of the developed pressure sensor, it was discerned to outperform existing piezoresistive‐based apparatuses significantly, as delineated in Figure 4 and Table 1. Notwithstanding, it is observed that conventional pressure sensors frequently encounter variations in sensitivity across distinct pressure levels—a phenomenon not entirely mitigated in this study. This limitation stems from the inherent restrictions in the deformation capacity and viscoelastic properties of the soft materials employed in the device's fabrication, culminating in a definitive threshold beyond which current alterations cease to escalate. This constraint complicates the accurate ascertainment of pressure magnitudes at elevated levels. To surmount this challenge, the incorporation of an AI model was proposed. In preparation for integrating the pressure sensor with a deep learning framework, current change signals were amassed under the application of a spectrum of 7 pressures ranging from 5 to 100 kPa, facilitating the training of a 1D‐CNN model with 1000 data instances per condition. **Figure** **8**a presents the schematic outline of the deep learning model's structure, illustrating the model's capacity to identify, extract, and assimilate patterns from the input data. The trained model adeptly classifies the data, distinguishing patterns by condition upon receiving arbitrary input values. Figure 8b,c displays the loss and accuracy functions of the model, revealing a trend where the loss diminishes and accuracy augments concomitantly with an increase in the number of learning epochs. The culminating test accuracy achieved was exceptionally high at 94.08%, indicating the model's proficiency in recognizing and categorizing the signal patterns for each condition with remarkable precision. Figure 8d showcases the derived confusion matrix, with all diagonal row values exceeding 0.89. This indicates that the model boasts a prediction accuracy surpassing 89% across all tested conditions, thereby evidencing its formidable performance. By amalgamating deep learning techniques, the pressure sensor heralds the feasibility of devising a system endowed with a sophisticated signal classification mechanism, marking a significant advancement in the realm of pressure sensing technology.

## 3 Conclusion

In conclusion, this investigation has successfully developed an advanced pressure sensor array employing MXene‐coated conductive fabrics and AgNW/polymer fiber‐based electrode assemblies, constituting a meticulously designed 10 by 10 array with 100 pixel sensors. Characterized initially by modest electrical conductivity owing to the sparse arrangement of conductive fibers, the sensor array exhibited a pronounced enhancement in current under applied pressure, demonstrating the critical role of the semi‐cylindrical electrode configuration in precisely determining the contact area upon force application, thus significantly improving sensitivity to levels as high as 888.79 kPa^−1^. The integration of components using reversibly cross‐linked PBUs and physical consolidation through heat treatment—excluding the AgNW and MXene layers encapsulating the polymer fibers—ensures that the sensor located between the electrode layers is immune to strain and remains undistorted within the array. This innovative approach delineates a paradigm for achieving both strain immunity and heightened sensitivity in stretchable piezoelectric pressure sensors, thereby setting a new benchmark in the field. A pivotal demonstration of this sensor array's capability was its ability to detect subtle airflow, as evidenced in the wind sensing test. An air gun emitting wind at 3 m s^−1^ from varying distances was used to illustrate the sensor array's sensitivity to slight air movement—a challenging feat for pressure sensors. The consistent detection of such minimal airflow underscores the exceptional performance and sensitivity of the developed sensor system. Furthermore, this study proposes a novel methodology to counteract the time‐dependent characteristic shifts or degradation inherent to polymer‐based pressure sensors, employing a deep learning algorithm. This technique significantly enhances the long‐term signal detection reliability of polymer‐based stretchable mechanical sensors, representing a considerable advancement in sensor technology. These results, along with the integration of deep learning for signal analysis, offer new insights into the potential applications of conductive fabrics and electrode shape deformation in sensor technology. They pave the way for innovative solutions that enhance the reliability and applicability of stretchy piezoelectric pressure sensors in real‐world scenarios, such as environmental monitoring and healthcare diagnostics, where detecting subtle changes in the environment is crucial.

## 4 Experimental Section

### Materials and Reagent

The titanium aluminum carbide (Ti_3_AlC_2_, 99.9%, 325 mesh) precursor was sourced from Carbon‐Ukraine. Chemical reagents such as hydrochloric acid (HCl, 37%), hydrofluoric acid (HF, 49%), lithium chloride (LiCl, 99.9%), methyl ethyl ketone (MEK), bis(3‐ethyl‐5‐methyl‐4‐maleimidophenyl)methane (BMI), isophorone diisocyanate (IPDI), and dibutyltin dilaurate were acquired from Sigma‐Aldrich, USA. Furfurylamine and glycerol 1,2‐carbonate were procured from Tokyo Chemical Industry, Japan. HLBH‐P 2000 was purchased from Cray Valley, USA. AgNWs, with an average diameter of 20 nm, an average length of 25 µm, an average aspect ratio of over 1000:1 dispersed in deionized (DI) water (0.5 wt.%), were prepared by C3 Nano, Hayward, Canada. Polydimethylsiloxane (PDMS, Sylgard 184) was sourced from Dow Corning, USA.

### Synthesis of PBU

Initially, diol **1** was synthesized through the reaction of glycerol 1,2‐carbonate (6.0 g, 50.8 mmol) with furfurylamine (4.9 g, 50.8 mmol), conducted over a duration of 3 h at a temperature of 60 °C. Subsequently, the resulting diol **1** (1.29 g, 6.0 mmol) was combined with HLBH‐P 2000 (12.0 g, 6.0 mmol), IPDI (2.67 g, 12.0 mmol), and MEK (15 g) to achieve a homogeneous mixture. This mixture was then subjected to further synthesis in an oil bath at 60 °C for 2 h. The procedural outline of this synthesis is depicted in Scheme S1 (Supporting Information).

### Synthesis of Ti_3_C_2_ MXene

A quantity of 1 g of Ti_3_AlC_2_ MAX precursor was gradually introduced into a solution comprised of DI water (12 ml), HCl (24 ml), and HF (6 ml) to facilitate the complete removal of the aluminum (Al) layer embedded within the MAX powder. This mixture underwent magnetic stirring at 500 rpm for 24 h at a temperature of 35 °C. Subsequently, 2 g of LiCl powder was added to the resultant mixture to initiate intercalation, followed by additional stirring at ambient temperature for 12 h. The final product was then subjected to repeated centrifugation at 3,500 rpm for 2 h to isolate the sediment, resulting in the dispersion of MXene nanosheets in DI water at a concentration of 0.8 wt.%.

### Fabrication of MXene/PBU‐Based Pressure Sensors

The PBU solution was loaded into a syringe preparatory for electrospinning, utilizing a high‐voltage electrospinning apparatus (ESR200R2, NanoNC, South Korea) for the process. The operational parameters were set to an applied voltage of 12 kV, a nozzle‐to‐ground distance of 150 mm, and a flow rate of 10 ml h^−1^. Subsequent to electrospinning, the PBU fibers were left to dry at ambient temperature for 30 min and then cured at 60 °C overnight to facilitate the DA reaction. Following curing, the PBU matrix underwent atmospheric O_2_ plasma treatment for 5 min (with an oxygen flow rate of 20 ml min^−1^ and power set to 150 W) and was then immersed in a 0.8 wt.% MXene nanosheets suspension. The matrix was agitated in the MXene suspension for 2 h, allowing the MXene nanosheets to infiltrate the matrix thoroughly. Afterward, the MXene/PBU composite was dried in a 60 °C oven for 1 h, with retro‐DA and DA reactions subsequently induced at 120 °C for 2 h and at 60 °C overnight, respectively. Extraneous MXene burrs on the PBU fiber surface were removed via ultrasonication for 10 min.

### Fabrication of AgNW/PBU‐Based Electrodes

To construct the individual PBU fibers, the PBU solution underwent electrospinning under specific conditions: an injection rate of 30 ml h^−1^, an applied voltage of 8 kV, and a distance of 20 mm between the nozzle and the ground. In preparation of the hydrophobic substrate, the PDMS base and curing agent were amalgamated in a 10:1 ratio, followed by spin‐coating at 1,000 rpm and subsequent curing at 100 °C. The PBU fibers were then cured at 60 °C overnight. Post‐curing, fibers pre‐strained to 30% elongation were arranged atop the PDMS substrate. AgNW solution was applied to the PBU fibers and allowed to dry at ambient temperature. Given that the PBU fiber exhibits a semi‐cylindrical form, meticulous care was taken during the fabrication process to ensure uniform coating across its surface. To achieve this, the entire substrate was systematically rotated multiple times during the application of coating materials. This rotational method was employed to mitigate the effects of gravitational forces that could potentially lead to uneven distribution of the coating substances. Such a procedure was critical in maintaining the consistency of the layer thickness and ensuring the functional integrity of the resulting sensor components. This careful handling was indicative of the rigorous standards adhered to in the manufacturing process, which were designed to overcome physical challenges and optimize product quality. This procedure was executed thrice, culminating in an AgNW/PBU electrode with a resistance of ≈0.8 ohm.

### Fabrication of Unit Sensors and 10 by 10 Sensor Matrix

Upon the meticulously prepared PBU film, sensor pixels were systematically deposited using a spin‐coating technique. Due to the effective grounding of the PBU during the electrospinning process, the bottom electrode exhibited a notably flat surface, facilitating its firm attachment to the PBU film. The MXene/PBU sensor pixels were precisely aligned at uniform intervals on this substrate. A top electrode was then positioned perpendicular to the bottom electrode across the sensor pixel array to establish a sandwich configuration. For the encapsulation layer, the PBU solution was electrospun across the entire substrate. During this phase, the concentration of the solvent MEK was adjusted to 25 g to refine the PBU synthesis process, aiming to produce fibers of smaller diameter. This adjustment was crucial for achieving a uniform thickness throughout the encapsulation layer. The specified conditions were meticulously maintained to support the activation of retro‐DA and DA reactions, which were critical for ensuring the structural integrity of the device upon thermal treatment.

## Conflict of Interest

The authors declare no conflict of interest.

## Supporting information

Supporting Information

## Data Availability

The data that support the findings of this study are available from the corresponding author upon reasonable request.
