# Supplementary material for: Stretchable Piezoresistive Pressure Sensor Array with Sophisticated Sensitivity, Strain‐Insensitivity, and Reproducibility
Source: Adv Sci (Weinh). 2024 Jul 16;11(35):2405374. doi: 10.1002/advs.202405374 (PMC11425275; doi:10.1002/advs.202405374)
Supplement: Supplementary file 1 — Supporting Information [file ADVS-11-2405374-s001.docx]

Copyright WILEY-VCH Verlag GmbH & Co. KGaA, 69469 Weinheim, Germany, 2013.

Supporting Information

Stretchable Piezoresistive Pressure Sensor Array with Sophisticated Sensitivity, Strain-Insensitivity, and Reproducibility

Su Bin Choi,^†^ Taejoon Noh,^†^ Seung-Boo Jung,^*^ Jong-Woong Kim^*^

^†^ These authors equally contributed to this work.

**Finite element analysis (FEA) for evaluating stress distribution**

Investigative analysis leveraging the finite element method (FEM) was undertaken to decipher the operational mechanism of our newly developed pressure sensor, focusing on stress distribution throughout the sensor and the surface area under applied weight. These analyses were conducted using the FEA feature in ANSYS 18.1 Static Structural. Additionally, the distribution of pressure across the surface area influenced by wind forces was examined through computational fluid dynamics (CFD) employing ANSYS 18.1 Fluent. The pressure sensor model was accurately designed to reflect its real-world dimensions, incorporating AgNWs (AgNWs)/polybutadiene-based urethane (PBU)-based electrodes, MXene/PBU-based pressure sensors, and an encapsulation layer. The material properties critical for the comprehensive three-dimensional FEM analysis are detailed in **Table S1**. Our simulation strategy entailed mimicking the load conditions on the sensor by applying gravitational forces akin to those exerted by objects resting on it, while the CFD analysis simulated wind conditions with a velocity of 3 m/s.

**Table S1**. The PBU properties employed in FEM.

| Property | Name | Value | Unit |
| --- | --- | --- | --- |
| Young’s modulus | E | 44.4 | kPa |
| Poisson’s ratio | Nu | 0.49 | 1 |
| Density | rho | 1252 | Kg/m^3^ |

**Characterization**

Field emission scanning electron microscopy (FESEM; SEMIRON 5000, SERON TECHNOLOGIES INC., South Korea), equipped with an energy dispersive X-ray spectroscopy (EDS) detector, was employed to scrutinize the surface morphology and chemical composition of the conductive fibers. The surface profiling of the AgNW layer structure was performed by using atomic force microscope (AFM; AFM5000, Hitachi High-Tech Scence Corporation, Japan). The phase and chemical composition of PBU was evaluated using X-ray diffraction (XRD; XRD-6100, Shimadzu, Japan) and X-ray photoelectron spectroscopy (XPS; ESCALAB250, Thermo Scientific, USA), respectively. Additionally, Fourier Transform Infrared (FT-IR) spectroscopy facilitated further elucidation of PBU's chemical properties. Mechanical properties were determined through the acquisition of a stress-strain curve utilizing a universal testing machine (34SC-1, Instron Corporation, USA). Electrical measurements, including resistance and current, were conducted using an LCR meter (LCR-6100, GwINSTEK, Taiwan) and an electrometer (Model 2461, Keithley, USA), respectively. The adhesion test between MXene and PBU was performed with Super Hold tape (1825D, 3M, USA).

**
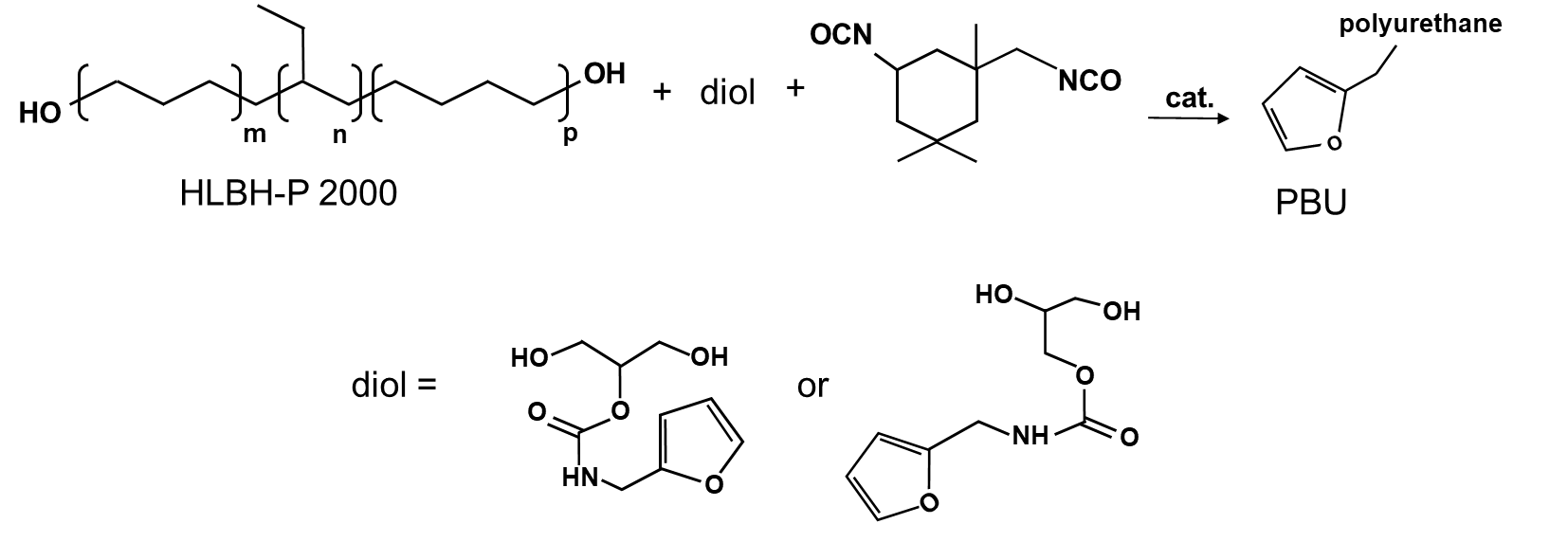
**

**Scheme S1**. Synthesis process of PBU.


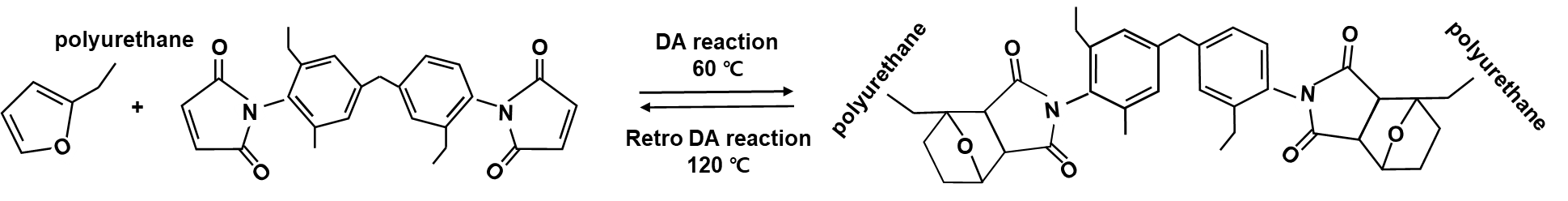


**Scheme S2**. Diels-Alder (DA) and retro-DA reactions of PBU.

**Figure S1.** Stress-strain curves of PBU film during cyclic tensile testing at 100% strain.

This figure presents the recorded stress-strain responses of PBU film subjected to cyclic tensile testing, where the material was repeatedly stretched to 100% of its original length. The curves illustrate the mechanical resilience and deformation characteristics of the PBU film over successive cycles, highlighting the material’s ability to withstand repetitive loading without significant loss of structural integrity or mechanical properties. Each curve represents one complete cycle, providing insights into the film’s elastic recovery and plastic deformation behaviors under continuous strain conditions.


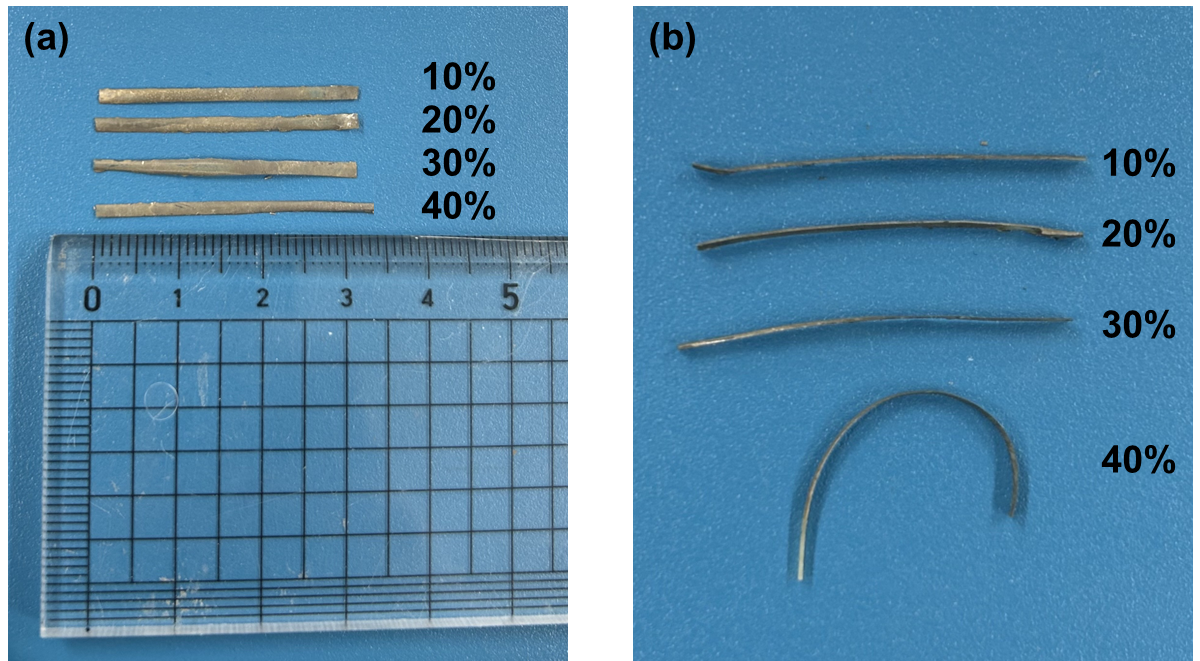


**Figure S2.** Digital images depicting the (a) top-view and (b) side-view of AgNW/PBU-based electrodes fabricated under pre-deformation conditions ranging from 10% to 40%.

The figure presents images of AgNW/PBU electrode samples subjected to varying degrees of pre-strain. Pre-strain levels ranging from 10% to 40% were applied to the samples. Notably, under the 40% pre-strain condition, the AgNW layer failed to fully withstand the compressive stress generated during the relaxation phase, resulting in the electrode not reverting to its original length. Additionally, as evidenced by the side-view image of the sample, the electrode did not restore to a flat configuration and instead exhibited an inward rolling deformation. These observations indicate that the maximum pre-strain that can be effectively applied to fabricate a strain-insensitive electrode is 30%. Beyond this threshold, the structural integrity of the AgNW layer is compromised, leading to deformation that undermines the electrode's functionality and performance.


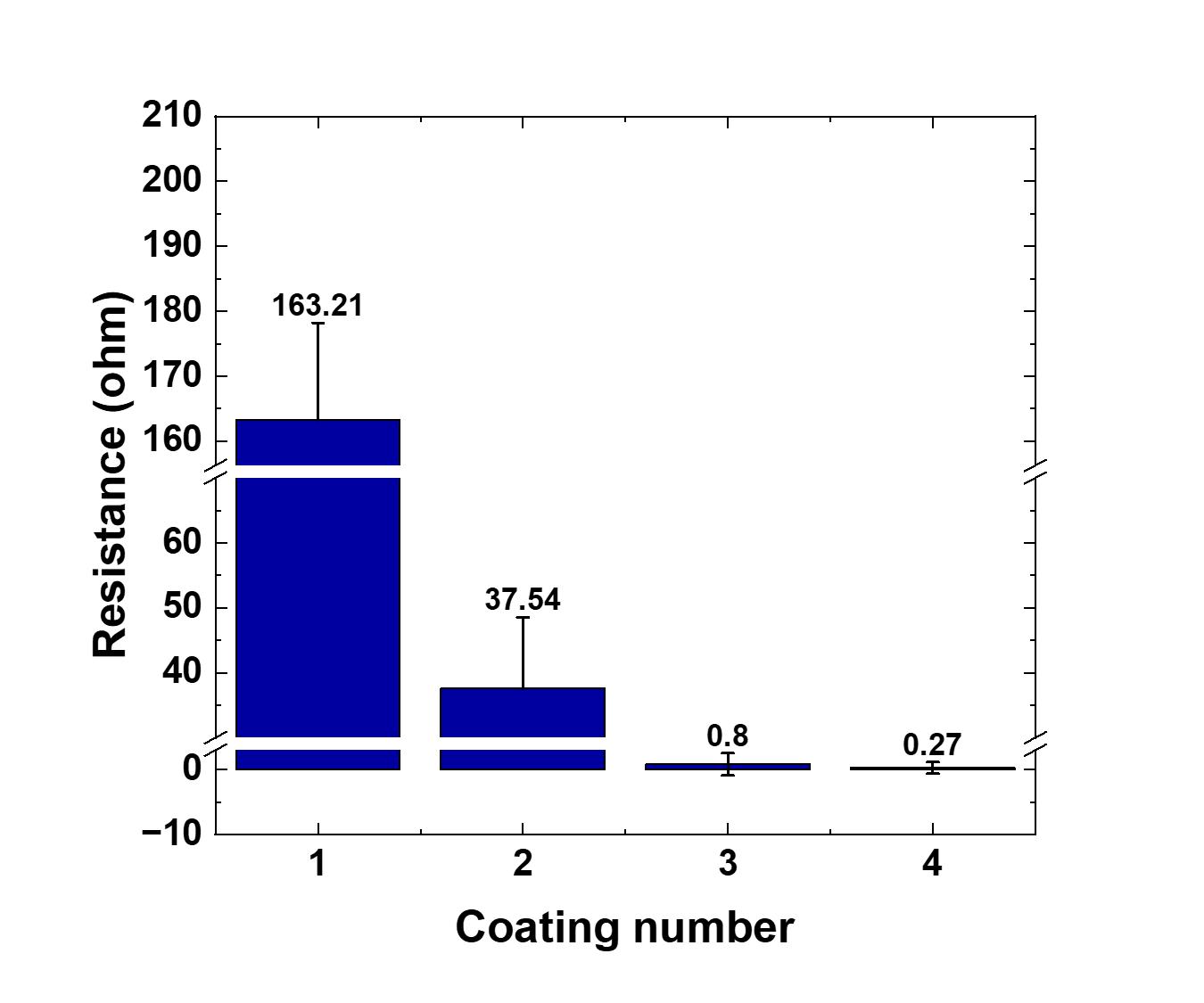


**Figure S3.** Electrical resistance of pre-strained PBU fiber post-release as influenced by the number of AgNW coatings.

This figure illustrates the correlation between the number of AgNW coatings applied to pre-strained PBU fibers and their subsequent electrical resistance after the fibers are released from stress. The graph demonstrates a clear trend where increasing the number of AgNW coatings results in a decrease in electrical resistance, indicating enhanced conductivity due to more extensive formation of conductive networks across the fiber surface. Each data point represents the average resistance measured across multiple samples for each coating level, reflecting the repeatability and reliability of the coating process in improving electrical properties. This systematic variation is crucial for optimizing the fiber's performance in applications where consistent electrical behavior under mechanical stress is required.


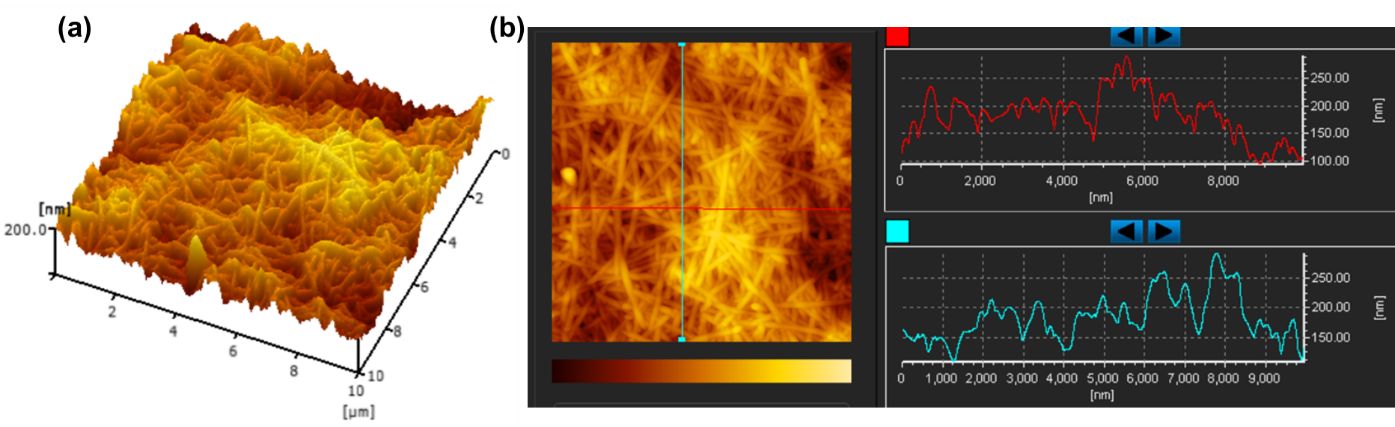


**Figure S4**. Detailed visualization of AgNW/PBU-based electrode structures: **(a)** This part of the figure displays three-dimensional surface scan images of the AgNW/PBU-based electrode, providing a comprehensive view of the topographical nuances of the electrode surface. These images highlight the intricate textural details and the uniform distribution of AgNW embedded within the PBU matrix, demonstrating the advanced fabrication techniques used to engineer these composite structures. **(b)** The height profiles corresponding to the scanned areas are shown, offering quantitative insights into the vertical dimensions of the surface irregularities and the distribution of material across the electrode. This analysis is crucial for understanding the physical characteristics of the electrode, such as thickness variations and the peak-to-valley measurements, which play significant roles in the electrode's functional performance in sensor applications.

**Figure S5**. Resistance variation in response to periodic stretching at 30% strain over 5000 cycles.


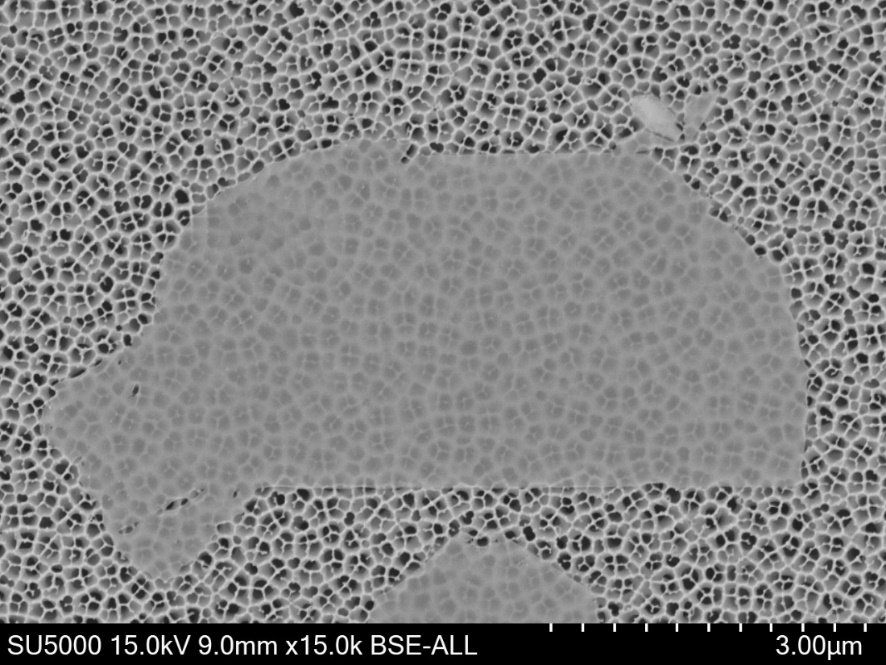


**Figure S6**. FESEM image of MXene nanosheet: A detailed visualization captured through FESEM, showcasing the intricate structure of a MXene nanosheet.


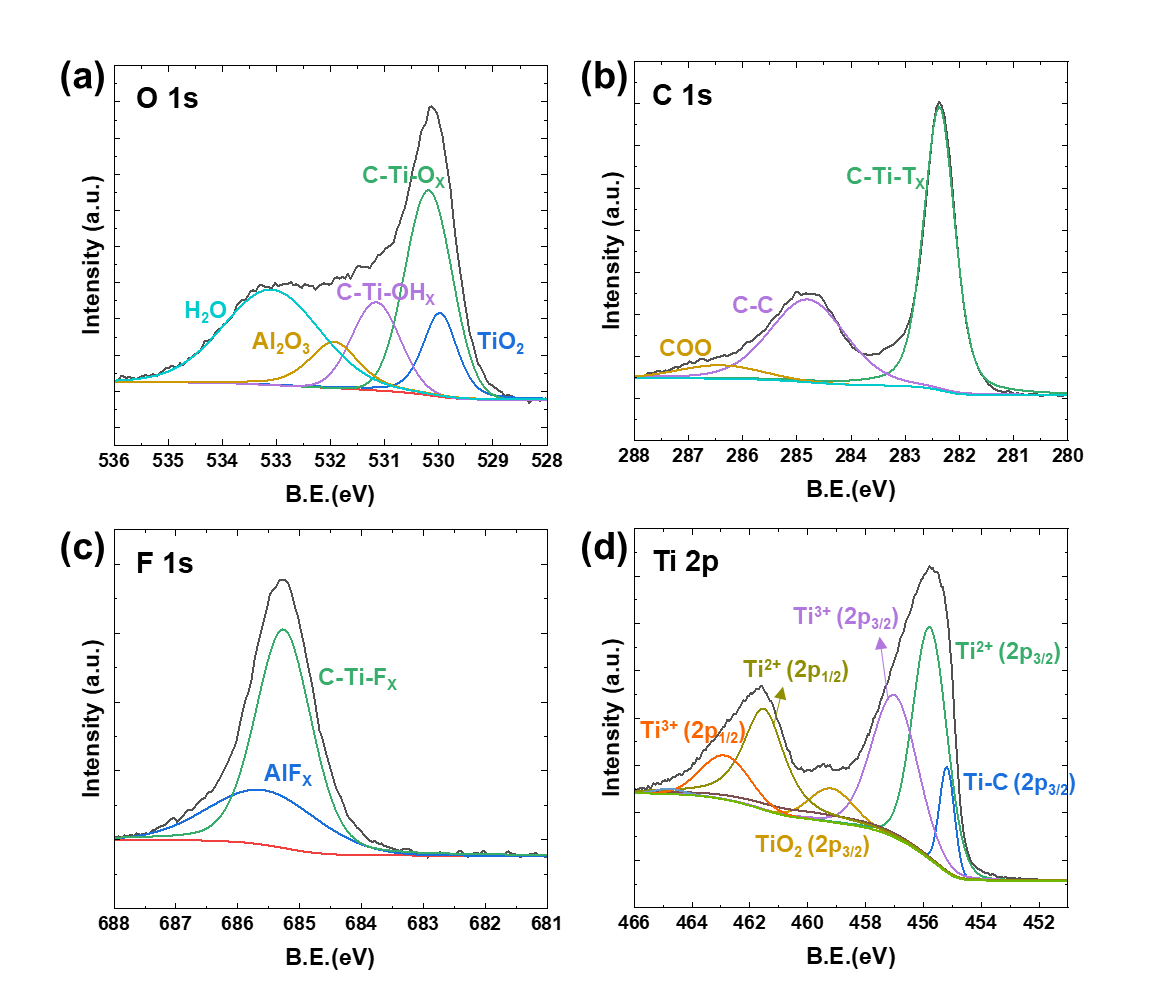


**Figure S7**. Deconvoluted XPS spectra analysis for Ti_3_C_2_-based MXene. Panels **(a)** O 1s, **(b)** C 1s, **(c)** F 1s, and **(d)** Ti 2p present the detailed XPS results, elucidating the chemical states and compositions of elements within the Ti_3_C_2_-based MXene.


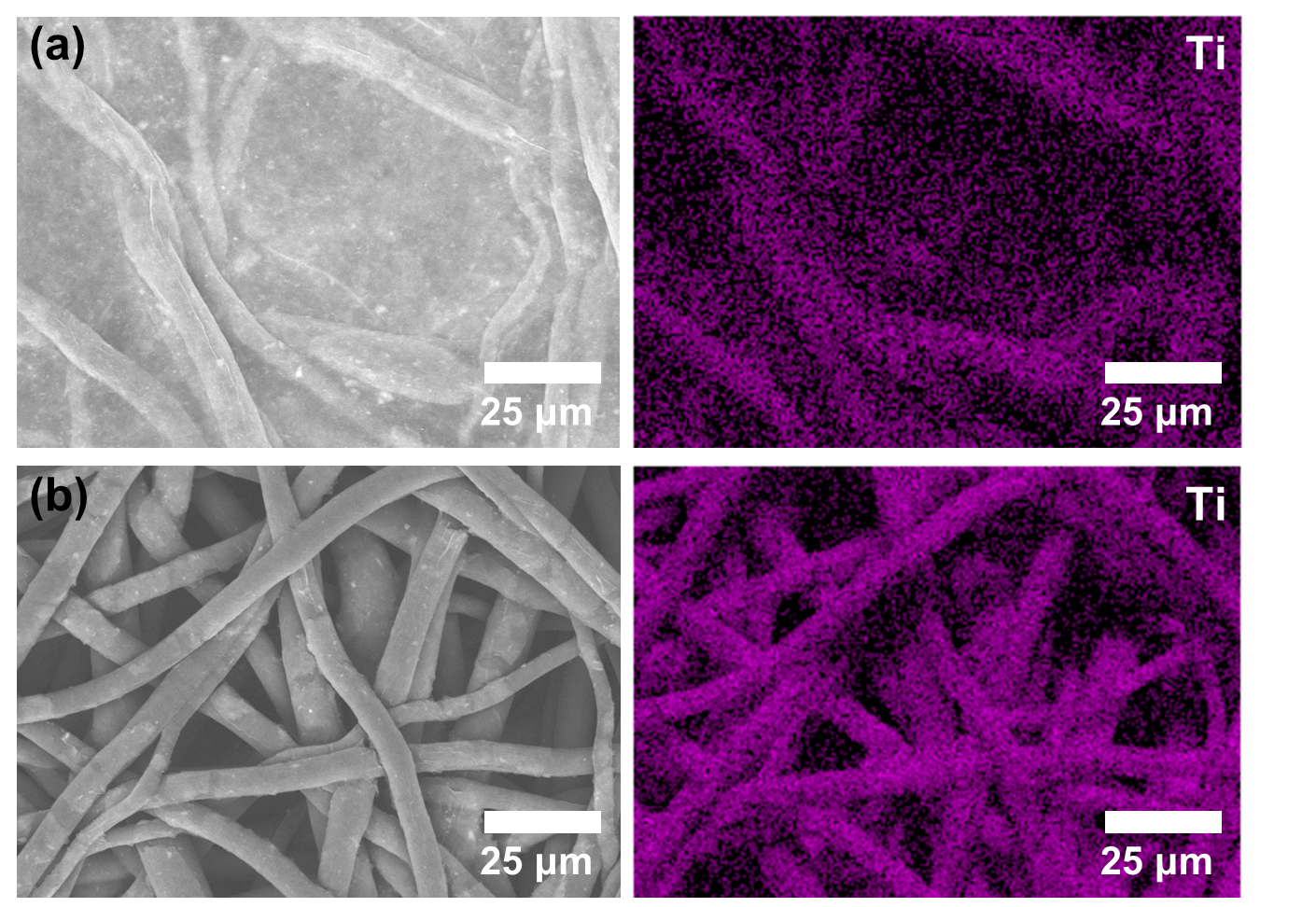


**Figure S8**. EDS elemental mapping of titanium in MXene-coated PBU. Panels **(a)** and **(b)** display the EDS mapping images of the titanium element within the MXene-coated PBU, before and after ultrasonication, respectively, illustrating the distribution and concentration changes.


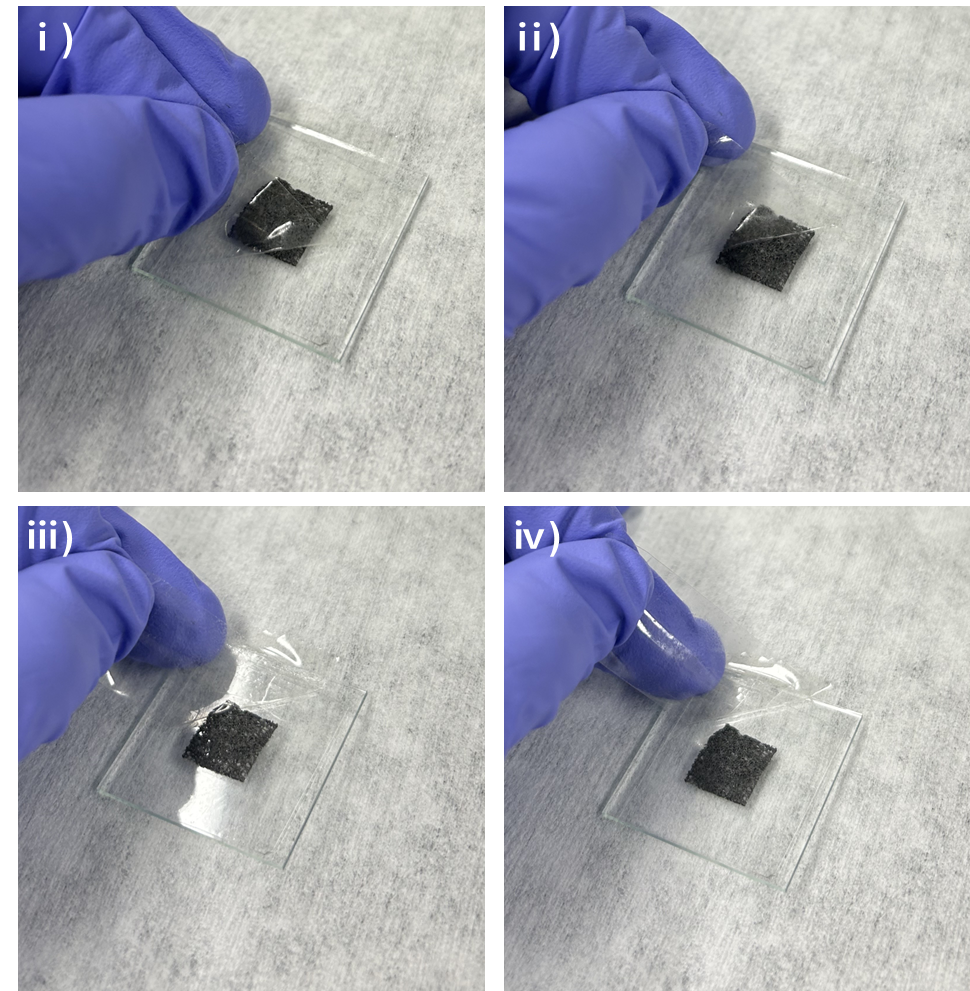


**Figure S9.** Sequential digital images illustrating tape detachment from the MXene-PBU composite matrix.

This series of images captures the critical steps involved in conducting the tape test to evaluate the adhesive strength and integrity of the interface between MXene layers and the PBU matrix. Each image details the process of applying and subsequently removing adhesive tape, a method used to assess the robustness of the bonding achieved through ultrasonic treatment. These visuals provide clear evidence of the MXene-PBU composite's resistance to mechanical peeling, showcasing the effective adhesion properties of the matrix under stress. This sequence is instrumental in demonstrating the composite's suitability for applications where mechanical durability and adhesive integrity are essential.


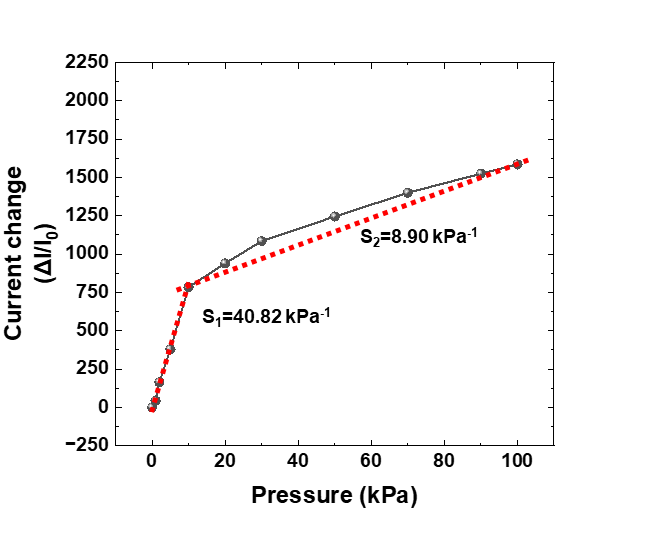


**Figure S10**. Sensitivity analysis of the pressure sensor equipped with a flat-shaped electrode. This figure presents the measured sensitivity of the pressure sensor when utilizing an electrode with a flat configuration, highlighting its performance under applied pressure.


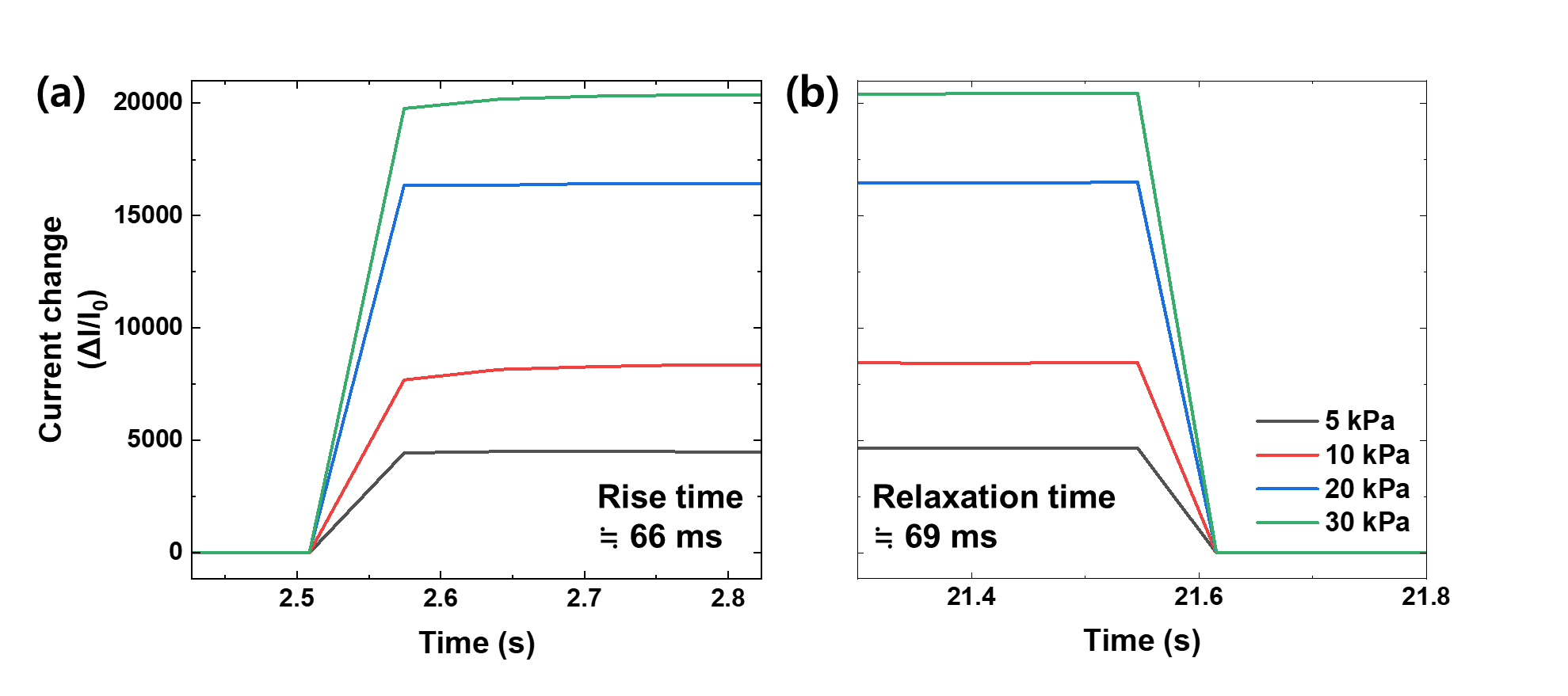


**Figure S11**. Response time analysis of the pressure sensor across a pressure range of 5 – 30 kPa. This figure depicts the pressure sensor's reaction time to varying pressures within the specified range, showcasing its operational speed and efficiency.

**Figure S12**. Current variation in strained AgNWs/PBU electrodes across a pressure spectrum of 1 to 100 kPa. This figure illustrates the changes in current observed in AgNWs/PBU electrodes subjected to strain, when exposed to pressures within the stated range, highlighting the electrodes' sensitivity and performance under different pressure levels.


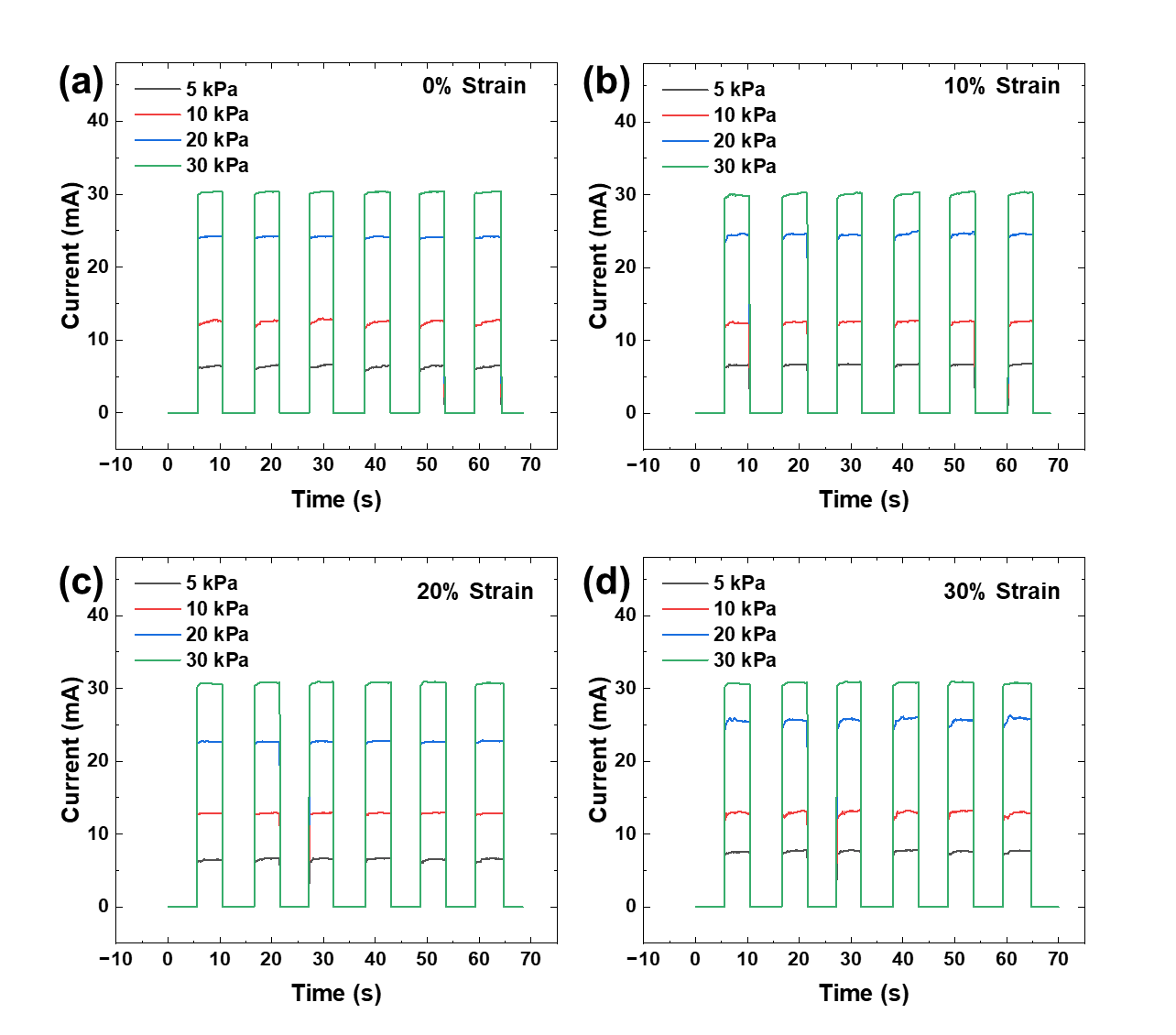


**Figure S13.** Pressure-dependent dynamic current curve under 0 - 30% stretch strain. Panels **(a)** to **(d)** correspond to **Figure 4(e)** to **(h)**, respectively.

**Figure S14.** Detailed record of current fluctuations during pressing-releasing durability test. This figure presents a comprehensive analysis of the rate of current changes observed in the sensor system during a rigorous pressing-releasing test conducted over 10,000 cycles, with a consistent applied pressure of 30 kPa. The data depicted provides insights into the sensor's electrical stability and reliability under repeated mechanical stress. Each data point represents the current response at a specific cycle, illustrating the sensor's ability to maintain consistent performance and signal integrity throughout the extended period of testing. The resilience of the sensor's electrical properties under such conditions underscores its suitability for applications demanding high durability and operational consistency.


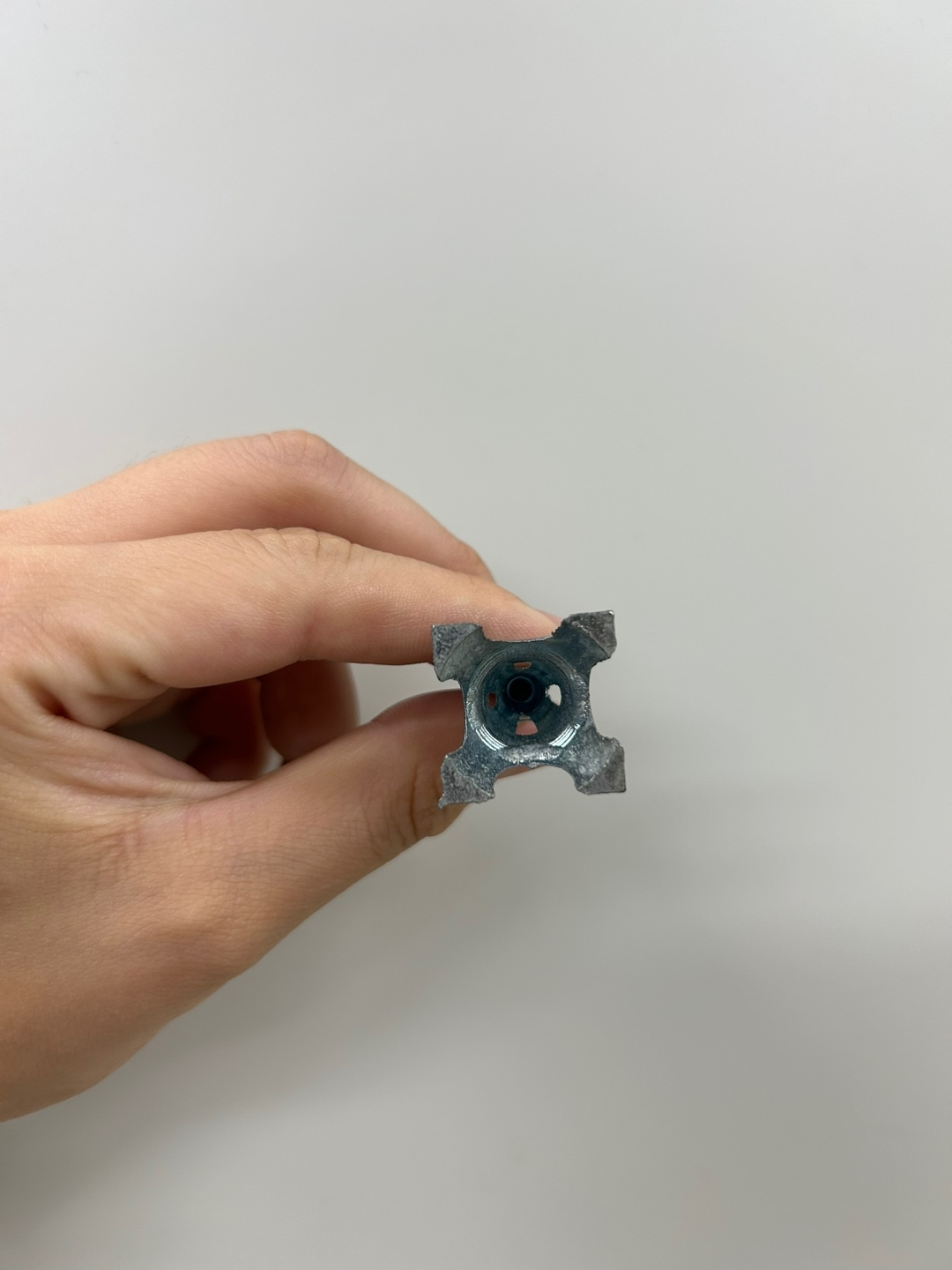


**Figure S15**. Digital image showcasing the bottom view of the Eiffel Tower model. This figure presents a visual depiction of the Eiffel Tower model's base, illustrating the structural details and design of the tower's lower section.


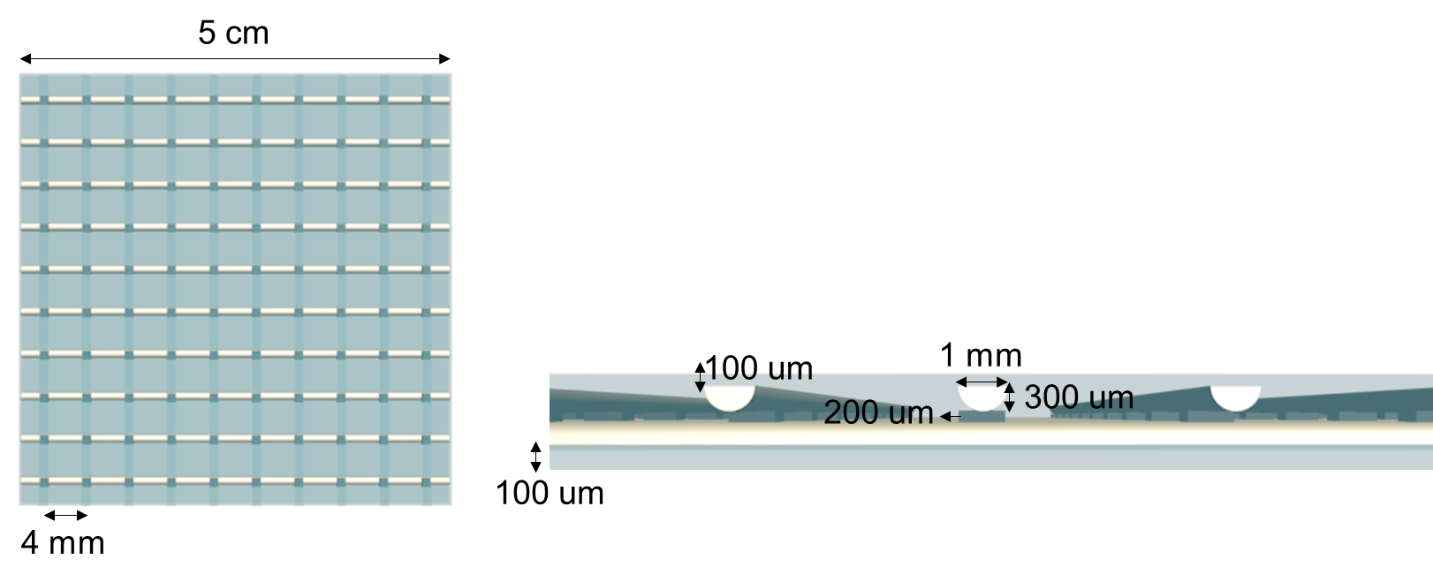


**Figure S16**. Technical schematic of the sensor array designed for simulation purposes. This figure provides a detailed technical drawing of the sensor array, outlining the dimensions and layout used in simulation studies, serving as a blueprint for the structural arrangement and component specifications.


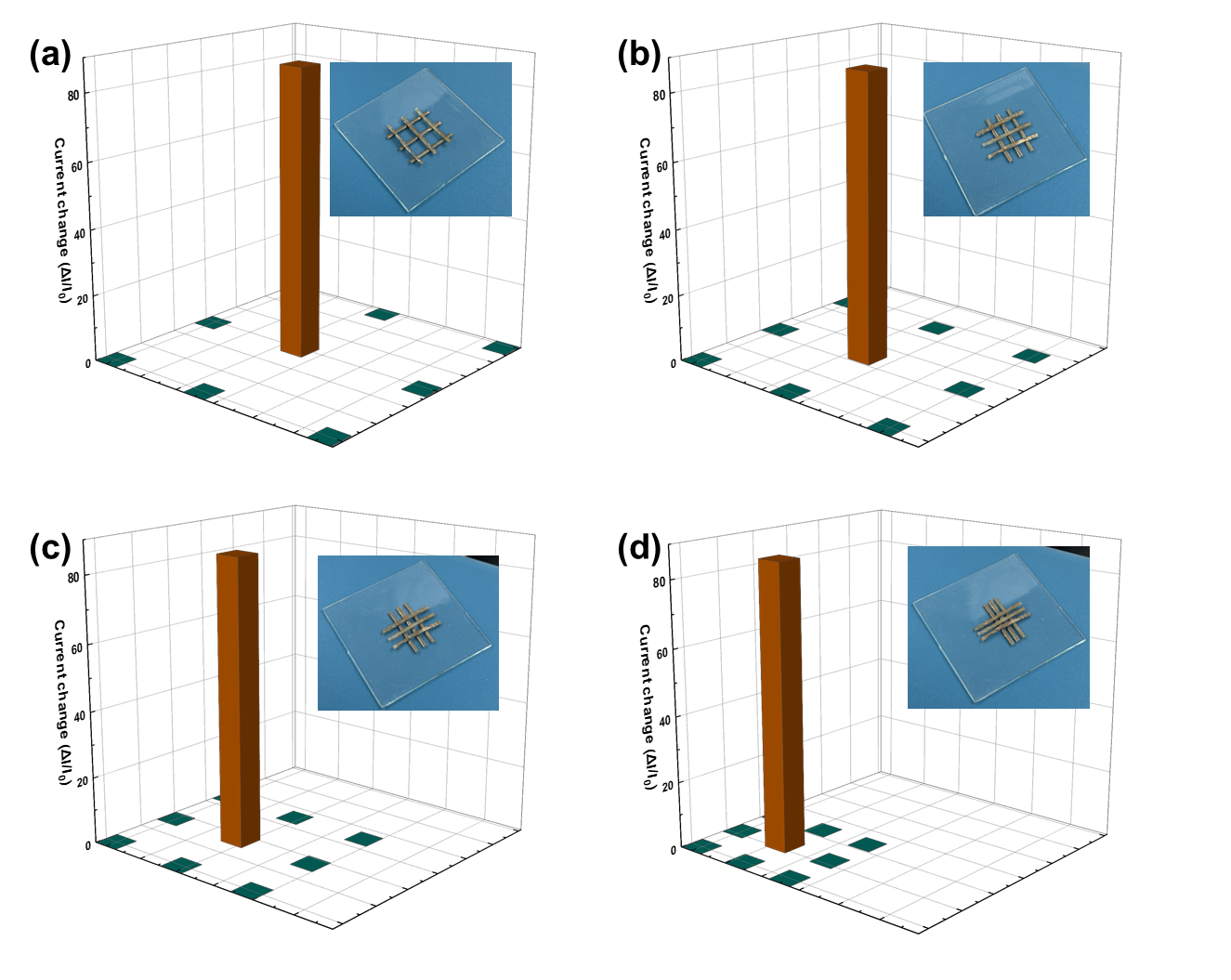


**Figure S17.** Output current distribution of the pressure sensor at varied sensor placement intervals. This chart illustrates the effects of sensor placement distance on the output current of the pressure sensor, with intervals set at **(a)** 1 mm, **(b)** 2 mm, **(c)** 3 mm, and **(d)** 4 mm. The data presented highlights how the spatial resolution and signal independence of sensors are maintained across these varying intervals, demonstrating minimal signal crosstalk even as the density of sensor placement increases.


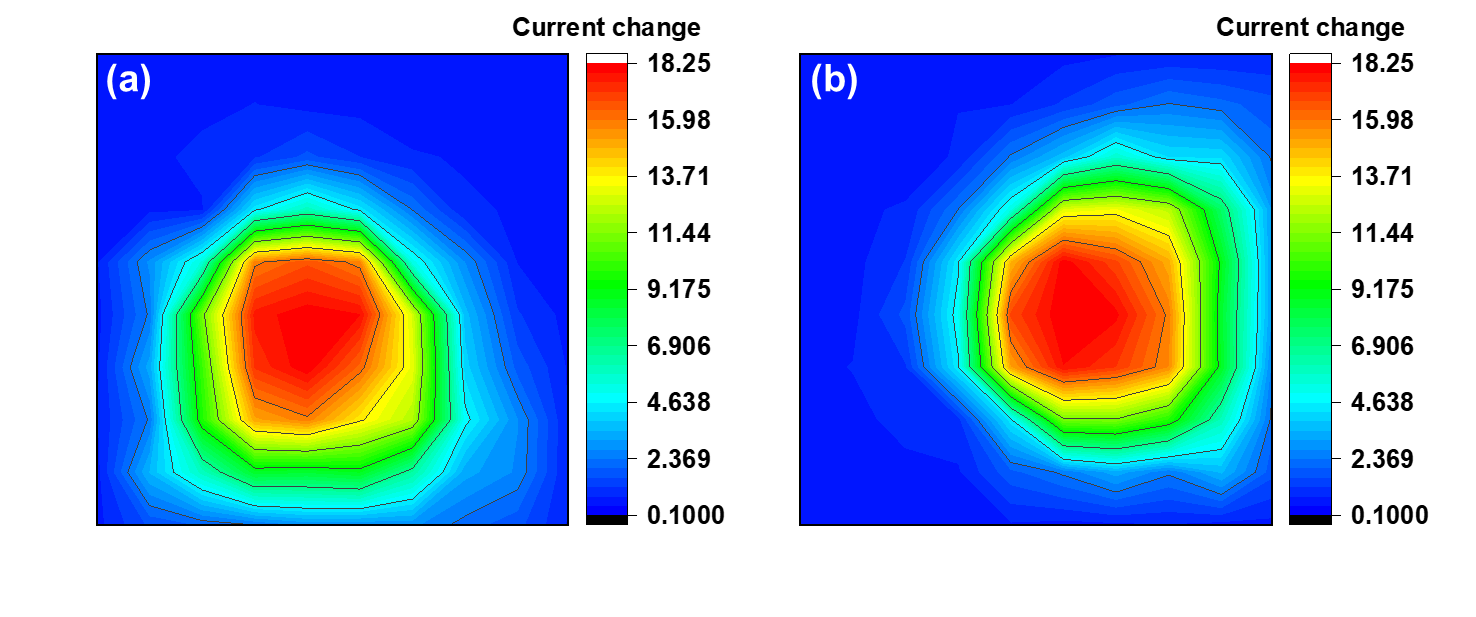


**Figure S18.** Detailed current distribution diagram reflecting varied angles of wind pressure source. This diagram illustrates the effects of changing the angle of the source of wind pressure on the current distribution across the sensor array. The source, positioned 20 mm away from the center of the array, is oriented to direct wind at a 45-degree angle toward both **(a)** north and **(b)** west relative to the array's central point. The figure visually represents how the orientation of the wind source influences the electrical responses detected by the sensors, with particular attention to the spatial variance in current intensity due to the directional wind flow. This setup allows for an in-depth analysis of the sensor array's sensitivity to changes in wind direction and the ability to pinpoint the origin of wind pressure based on current fluctuations within the array.
